# Advances in the use of entomopathogenic fungi to control ixodid ticks

**DOI:** 10.1590/S1984-29612026004

**Published:** 2026-04-20

**Authors:** Patrícia Silva Golo, Jéssica Fiorotti, Isabele da Costa Angelo, Allan Felipe Marciano, Mariana Guedes Camargo, Caio Marcio de Oliveira Monteiro, Wendell Marcelo de Souza Perinotto, Éverton Kort Kamp Fernandes, José Reck, Vânia Rita Elias Pinheiro Bittencourt

**Affiliations:** 1 Universidade Federal Rural do Rio de Janeiro – UFRRJ, Instituto de Veterinária, Departamento de Parasitologia Animal, Seropédica, RJ, Brasil; 2 VITTIA Defesa e Nutrição, São Joaquim da Barra, SP, Brasil; 3 Universidade Federal Rural do Rio de Janeiro – UFRRJ, Instituto de Veterinária, Departamento de Epidemiologia e Saúde Pública, Seropédica, RJ, Brasil; 4 Lallemand Plant Care Brazil, Regulatory Affairs, Piracicaba, SP, Brasil; 5 Universidade de Vassouras, Fundação Severino Sombra – FUSVE, Vassouras, RJ, Brasil; 6 Universidade Federal de Goiás – UFG, Instituto de Patologia Tropical e Saúde Pública – IPTSP, Goiânia, GO, Brasil; 7 Instituto de Pesquisas Veterinárias Desidério Finamor – IPVDF, Eldorado do Sul, RS, Brasil

**Keywords:** Mycoacaricides, integrated pest management, Rhipicephalus microplus, Metarhizium, Beauveria, microbial control, Micoacaricidas, manejo integrado de pragas, Rhipicephalus microplus, Metarhizium, Beauveria, controle microbiano

## Abstract

Ticks are obligate blood-feeding ectoparasites of major veterinary and medical importance, and their control relies heavily on synthetic acaricides, a strategy increasingly compromised by resistance and environmental concerns. Entomopathogenic fungi (EPF) have gained significant prominence as biological alternatives for integrated tick management. This review synthesizes over two decades of advances in fungal-based tick control, including recent field trials, formulation technologies, and omics-driven mechanistic insights. Evidence shows that species within the genera *Metarhizium* and *Beauveria* exhibit consistent pathogenicity against several ixodid ticks, although efficacy varies with fungal isolate, propagule type, formulation, and environmental conditions. Advances in omics approaches have improved understanding of infection mechanisms and tick immune responses, while formulation and delivery innovations have enhanced fungal stability and field persistence. Field studies demonstrate variable but promising efficacy, particularly when EPF are applied within integrated control strategies. However, important challenges remain, including limited large-scale field validation and optimization of formulations. By integrating laboratory, field, and commercial evidence across production systems, this review identifies key technological bottlenecks and highlights future directions for climate-resilient and sustainable tick control.

## Resumo

Carrapatos são ectoparasitos hematófagos obrigatórios de grande importância veterinária e médica, e seu controle é feito através de carrapaticidas sintéticos, uma estratégia cada vez mais afetada pela resistência e por preocupações ambientais. Os fungos entomopatogênicos (FEP) destacam-se significativamente como alternativas biológicas para o manejo integrado de carrapatos. Esta revisão sintetiza mais de duas décadas de avanços no controle de carrapatos baseado em fungos, incluindo ensaios de campo recentes, tecnologias de formulação e insights mecanísticos impulsionados por ômicas. Evidências demonstram que espécies dos gêneros *Metarhizium* e *Beauveria* apresentam patogenicidade consistente contra diversas espécies de ixodídeos, embora a eficácia varie de acordo com o isolado fúngico, tipo de propágulo, formulação e condições ambientais. Avanços em abordagens ômicas ampliaram a compreensão dos mecanismos de infecção e resposta imune dos carrapatos, enquanto inovações em formulação e sistemas de aplicação têm melhorado a estabilidade e persistência dos fungos em campo. Estudos de campo demonstram eficácia variável, porém promissora, especialmente quando os FEP são aplicados em estratégias de controle integrado. No entanto, desafios importantes permanecem, incluindo a limitada validação em larga escala e a necessidade de otimização das formulações. Ao integrar evidências laboratoriais, de campo e comerciais em diferentes sistemas produtivos, a presente revisão identifica gargalos tecnológicos e destaca direções futuras para um controle de carrapatos sustentável e resiliente.

## Introduction

Ticks are obligate hematophagous ectoparasites that parasitize a wide range of vertebrate hosts, including mammals, birds, amphibians and reptiles. The order Ixodida comprises three recognized families: Argasidae, Nuttalliellidae, and Ixodidae. Among them, Ixodidae is distinguished by the presence of a rigid dorsal shield (scutum), a morphological trait that sets them apart from the other tick families. Their life cycle comprises four stages (egg, larva, nymph, and adults), with each parasitic stage requiring a blood meal to develop or reproduce (Sonenshine & Roe, 2014). Prolonged attachment and feeding are facilitated by specialized morphological and biochemical adaptations, such as a barbed hypostome, cement-like secretions, and salivary molecules with anticoagulant, anti-inflammatory, and immunomodulatory properties (Šimo et al., 2017). These adaptations enable ticks to remain undetected by the host immune system while ensuring efficient acquisition of nutrients essential for survival. According to Guglielmone et al. (2023), Ixodidae currently comprises 762 species worldwide. Beyond their role as parasites, ixodid ticks are of major veterinary and medical importance as vectors of a wide array of pathogens, including *Borrelia burgdorferi*, *Babesia* spp., *Anaplasma* spp., *Theileria* spp., *Rickettsia* spp., and tick-borne encephalitis virus (De la Fuente et al., 2008). Consequently, they influence host health, livestock productivity, and ecosystem dynamics through their impact on host populations and host-pathogen interactions.

Contemporary tick control relies predominantly on chemical acaricides (viz., synthetic pyrethroids, organophosphates, amidines, macrocyclic lactones, benzoylphenylureas, phenylpyrazoles and isoxazolines) delivered through dip vats, sprays, injectables and pour-on formulations (Makwarela et al., 2025). This chemical dependency represents a fundamental shift from pre-1960s genetic resistance strategies to widespread chemotherapeutic reliance (FAO, 2025). The use of chemical acaricides has made a considerable contribution to tick control; however, the exclusive reliance on this method has become unsustainable in the long term. Many active molecules have been lost due to the selection of resistant populations, and the discovery/production of new compounds is becoming increasingly challenging. Intensive acaricide use proves deleterious across multiple domains (Klafke et al., 2024). For animal health, chemical residues accumulate in livestock tissues while disrupting beneficial dung beetle populations essential for pasture ecology. Human health risks include occupational exposure during application and potential food residue consumption (De Meneghi et al., 2016). Environmental contamination affects aquatic ecosystems and non-target invertebrates through agricultural runoff (Rahman et al., 2022). Economically, the $8 billion global parasiticide market reflects substantial farm expenditures, with resistance development forcing increased application frequencies and higher costs (Pérez-Otáñez et al., 2024). The emergence of widespread acaricide resistance across major chemical classes validates concerns about unsustainable chemical dependency, necessitating integrated approaches combining genetic selection strategies, pasture management, vaccines, botanical alternatives, and the use of biological control.

The biological control of arthropod pests, defined as the use of natural enemies (viz., predators, parasites, parasitoids, and pathogenic microorganisms) to regulate pest populations and maintain ecological balance, has its origins in the earliest records of insect diseases. According to Steinhaus (1975), reports of diseases in bees date back to around 700 B.C. In Europe, the death of silkworms prompted extensive studies aimed at combating “muscardines,” caused by fungi. This historical recognition that microorganisms could infect and kill insects led humans to intentionally use these pathogens to control pest populations in crops. The use of entomopathogenic microorganisms as biocontrol agents of arthropods is an attractive and sustainable tool for many integrated pest management programs in forestry, agricultural, and veterinary settings.

Entomopathogenic fungi (hereafter referred to as EPF) infect hosts by penetrating the cuticle, proliferating in the hemocoel, and ultimately killing them through nutrient depletion and toxin production. Building on their successful use against insect pests, EPF have emerged as promising biological control agents for ticks (Camargo et al., 2014, 2016; Marciano et al., 2021) particularly in the face of growing acaricide resistance in tick populations (Klafke et al., 2024). EPF are generally considered safe for application in agricultural systems; currently, 283 fungal-based biopesticide products are registered in Brazil (Mascarin et al., 2024), reflecting their regulatory acceptance and widespread use.

The present article aims to provide a comprehensive overview of key aspects of EPF use for tick control, including diversity of EPF species; tick-fungal-pathogen interactions; microbiome-mediated interactions; formulation technologies and delivery systems; integration with chemical acaricides; field applications and real-world efficacy; regulatory framework and safety assessment; and knowledge gaps and future research directions. Unlike most reviews, which tend to focus on specific technical aspects, this work adopts a broader and integrative perspective to advance the state of the art and contribute to ongoing discussions surrounding the regulatory framework for the use of EPF against ticks, with particular focus on Brazil.

## Fungal Species Diversity and Strain Selection

EPF are phylogenetically diverse microorganisms that infect and kill not only insects, as this term indicates, but also several other invertebrate species, including ticks and mites. Entomophthoramycota is the phylum of the Kingdom Fungi with the greatest number of entomopathogenic species (Mora et al., 2017), although most mycopesticides have hypocrealean fungi (Ascomycota, Hypocreales) as active ingredients, including the key genera *Metarhizium*, *Beauveria*, *Hirsutella*, *Akanthomyces* (formerly, *Lecanicillium*), and *Cordyceps* (formerly, *Isaria*) (Faria & Wraight 2007; Mascarin et al., 2015, 2024). While most mycopesticides commercially available are based on aerial conidia produced by solid-state fermentation, submerged fungal propagules (e.g., blastospores, submerged conidia, and microsclerotia), produced by liquid fermentation, are also potential active ingredients. Other fungal propagules, such as mycelium or chlamydospores, however, are less promising for pest control (Mascarin et al., 2024). In fact, there are a few mycopesticides based on liquid-grown propagules available on the market, primarily from *Cordyceps fumosorosea*, *Cordyceps javanica*, and *Akanthomyces muscarius*, and none of them are available in Brazil (Mascarin et al., 2024).

Most studies have investigated the effects of aerial conidia of EPF, especially those from the *Metarhizium anisopliae* and *Beauveria bassiana* complexes, against ticks under laboratory conditions or in field trials (Fernandes & Bittencourt, 2008). More recently, the activity of fungal blastospores (Bernardo et al., 2018; Marciano et al., 2021) and microsclerotia (Marciano et al., 2021; Paixão et al., 2023) towards ticks has been examined with promising results. Blastospores, like conidia, are infective propagules that invade their hosts by penetrating the cuticle (Bernardo et al., 2018). Microsclerotia, however, are not directly infective propagules to ticks, but they produce *in situ* infectious spores under optimum conditions. Due to this condition, microsclerotia have been formulated in granules and applied against ticks off the host, in infested pastures (Marciano et al., 2021; Filgueiras et al., 2025). In fact, those fungal propagules have both advantages and disadvantages as biocontrol agents against ticks, and further investigations are needed to identify the most effective strategies for targeting the host at specific developmental stages or environments.

Fungal isolates of the same EPF species may exhibit high genetic diversity, high variability in virulence to ticks or in tolerance to environmental factors (Fernandes et al., 2010, 2011). Also, different propagules of the same fungal isolate may exhibit different efficacy against ticks or variable tolerance to environmental factors, such as high temperatures or ultraviolet radiation (Bernardo et al., 2018, 2020; Corval et al., 2021). In fact, a substantial phenotypic plasticity is found in isolates of EPF (Rangel et al., 2015). This refers to the remarkable ability of the same fungal isolate, with a given genotype, to exhibit different characteristics (phenotypes) in response to variations in environmental conditions, which may include nutrient availability and physical and chemical stresses (Rangel et al., 2015). Therefore, studies on screening for promising fungal strains, optimizing culture media, and enhancing appropriate formulation techniques are crucial for developing effective bioinputs. While the future of submerged liquid fermentation technology for the mass production of entomopathogenic fungi remains uncertain, it is anticipated to hold significant promise (Mascarin et al., 2024).

## Tick-fungal Pathogen Interactions: Recent Advances

### Infection process

The infection process of EPF in ticks involves a series of coordinated stages, including host recognition, conidial adhesion to the cuticle, germination, active penetration, internal colonization, and sporulation after the host’s death (Beys-da-Silva et al., 2020; Hong et al., 2024). Emerging evidence indicates that, prior to successful cuticle penetration, EPF must outcompete the host-associated ectomicrobiota and overcome or evade the antifungal immune defenses (Hong et al., 2024). Infection begins when airborne conidia reach the surface of the tick cuticle, a process facilitated by hydrophobic interactions (Ortiz-Urquiza & Keyhani, 2013). Studies with insects reported that this adhesion is primarily mediated by surface proteins known as hydrophobins (Skinner et al., 2014) and adhesins (Valero-Jiménez et al., 2016). Different types of hydrophobins provide the hydrophobicity required for the inoculum to interact with the arthropod’s integument (Butt et al., 2016; Jiang et al., 2020). Other proteins, such as CWP10, MAD1, and MAD2 (adhesin-like proteins) (Li et al., 2010; Abro et al., 2019; Zhou et al., 2021), are involved in conidial adhesion to the cuticle, participating in the initial recognition of the host in multiple EPF species and influencing germination, blastospore formation, and virulence (Wang & St. Leger, 2007; Gao et al., 2020; Zhou et al., 2021).

The fungal lipolytic activity observed in infected ticks, mediated by enzymes such as lipases and esterases, contributes to the process of conidial recognition and attachment, playing an essential role in the adhesion of *Metarhizium* to the cuticle of *Rhipicephalus microplus* (Santi et al., 2010; Usman et al., 2024). Once firmly adhered to the cuticle, the conidia start the germination phase, which depends on favorable environmental conditions (i.e., optimal temperature and humidity), producing a germ tube that gives rise to the appressorium or penetration peg, structures that enable active traversal of the cuticle (Brunner-Mendoza et al., 2019). The distinct lipid composition of the cuticle among tick species is a factor that interferes with the germination process (Ribeiro-Silva et al., 2022) and the subsequent penetration, which depends on the coordinated action of hydrolytic enzymes such as chitinases, lipases, and proteases, as well as the mechanical force exerted by the appressorium (Brunner-Mendoza et al., 2019). Each cuticular layer presents distinct polymeric compositions that are sequentially degraded by proteases and chitinases (Santi et al., 2010). Several proteases participate in this process, including chymotrypsins, metalloproteases, trypsins, exopeptidases, subtilisins, and aspartyl-peptidases (Semenova et al., 2020). The expression of these enzymes is modulated by the specific cuticular composition of each tick species (Freimoser et al., 2005); in *Metarhizium* sp. and *Beauveria* sp., up to 11 different subtilisins can be produced, with Pr1-type peptidases being particularly important for the pathogenicity of these fungi against arthropods. In addition to promoting cuticular degradation, these proteases contribute to nutrient acquisition and the success of host invasion (Gao et al., 2020; Semenova et al., 2020). Complementarily, Lu et al. (2024) demonstrated that the coordinated expression of hexosaminidases and chitinases is a key determinant of fungal pathogenicity, as it promotes cell wall remodeling, facilitates evasion of host immune responses, and enhances insect cuticle degradation, ultimately increasing fungal virulence.

After crossing the cuticular barrier, the fungus colonizes the hemocoel and differentiates into hyphal bodies, which are specialized propagules adapted to colonize the host’s internal environment (Pendland et al., 1993), or into analogous structures known as blastospores when produced *in vitro* (Mascarin et al., 2015). These structures spread throughout the tick’s body via hemolymph circulation and invade various tissues (Maina et al., 2018; Brunner-Mendoza et al., 2019). During this phase, EPFs employ multiple virulence factors that ensure their proliferation and the consequent death of the host. Among the most relevant are mycotoxins produced during fungal growth, such as beauvericin, beauverolides, oosporeins, and destruxins, which act as potent toxic compounds targeting ticks (Pedras et al., 2002; Maina et al., 2018; Pedrini, 2022).

These toxins disrupt cellular and structural processes, impair calcium channels, and cause flaccid paralysis, morphological alterations in muscular tissues, and damage to the Malpighian tubules, midgut, and other organs (Mora et al., 2017). Beauvericin, a cyclic hexadepsipeptide belonging to the enniatin family, is particularly well studied and has been identified in *Beauveria bassiana* and *Fusarium* (Hamill et al., 1969; Logrieco et al., 1998). In addition to its insecticidal activity, it exhibits antiviral, antibacterial, and antifungal properties, enhancing the efficacy of conventional antifungal drugs such as ketoconazole and miconazole (Zhang et al., 2007) and even demonstrating antitumor and antiviral activity in human models (Wang & Xu, 2012).

Following host death and nutrient depletion, the fungus breaks through the integument, forming aerial mycelium and initiating sporulation on the cadaver, which enables conidial dispersal and the infection of new hosts (Valero-Jiménez et al., 2016). The infection mechanism is also influenced by immune responses, as will be discussed in section 3.b. A deeper understanding of the molecular mechanisms involved in each stage of infection, as suggested by Beys-da-Silva et al. (2020), requires a transdisciplinary approach that integrates genomics, transcriptomics, proteomics, and metabolomics.

### Immune response

Ticks are invertebrate animals and do not have an adaptive immune system and, for this reason, defense against pathogens such as EPF occurs through the innate response. The cellular immune response takes place immediately after the invasion of microorganisms and is mediated by hemocytes, cells circulating in the hemolymph, through processes such as phagocytosis, encapsulation, and nodulation (Sterba et al., 2011; Tan et al., 2013; Fiorotti et al., 2022). The humoral immune response, in turn, involves various antimicrobial peptides and enzymatic cascades that regulate coagulation and hemolymph melanization. It may also involve the production of soluble molecules such as pathogen recognition proteins, thioester-containing proteins (TEPs), and reactive oxygen species (ROS) (Vass & Nappi, 2001; Kopácek et al., 2010; Smith; Pal, 2014).

A type of immune response that mimics memory in vertebrates is known as immune priming and occurs when a prior infection experience leads to a more effective response upon secondary exposure to the infection or pathogen (Bozler et al., 2020). Regarding immune priming against EPF, it has been demonstrated that there is no evidence that *Rhodnius pallescens* obtains a benefit in terms of survival because of an immune priming response after exposure to a nonlethal dose of the pathogenic fungus *B. bassiana* (Galvez et al., 2024) and there is also no evidence that ticks respond to EPF throughout immune priming.

#### Cellular immune response

The innate cellular immune response is primarily mediated by hemocytes, cells circulating in the hemolymph that can also be found adhered to the fat body and salivary glands (Sterba et al., 2011).

**Figure 1:**
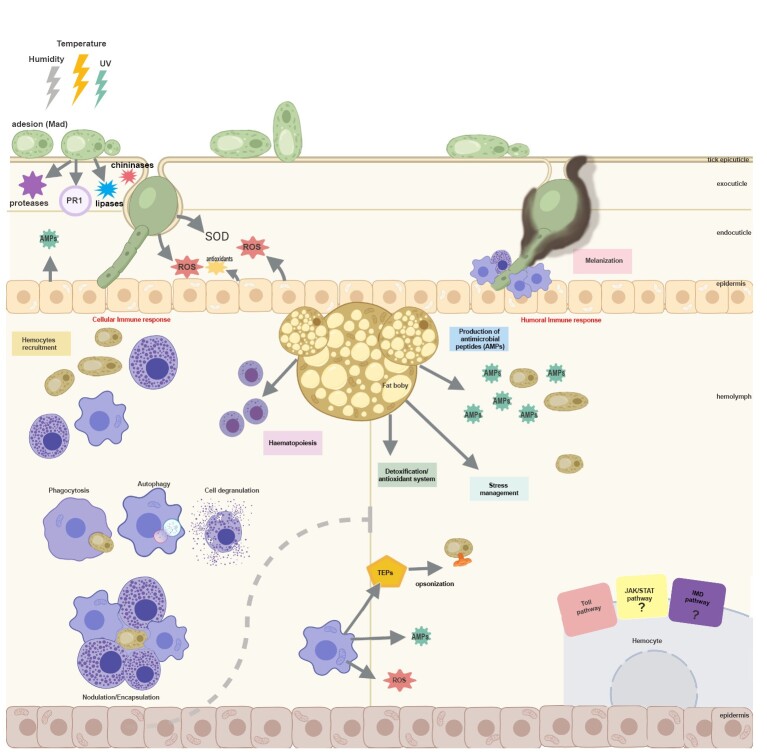
Overview of tick immune system responses to entomopathogenic fungal infection. *Metarhizium* is primarily exposed to stress factors such as temperature, UV radiation, and humidity. After adhesion of the entomopathogen (mediated by the Mad gene), germ tube and appressorium formation occurs. For the penetration process, the fungus must degrade the cuticle, mainly through PR1 (protease), in addition to releasing other proteases, lipases, and chitinases. Fungi may also exert mechanical pressure to penetrate the tick host while the host is already beginning to respond to infection. Subsequently, the tick mounts a cellular immune response, with hemocyte recruitment to the infection site, triggering processes such as phagocytosis, autophagy, and nodulation/encapsulation. The fat body plays a central role in immunity, being responsible for hematopoiesis, antimicrobial peptide (AMP) production, detoxification, and oxidative stress control. Moreover, hemocytes may release thioester-containing proteins to opsonize the pathogen for phagocytosis, and AMPs and reactive oxygen species are released by hemocytes. When the cellular response fails to overcome the infection, the humoral response becomes crucial, occurring through AMP release and activation of the Toll and the IMD pathways, as well as stimulation of the melanization process, in which hemocytes are also recruited.

Using RNA interference (RNAi), it has been shown that phagocytosis of *Candida albicans* by hemocytes of the tick *Ixodes ricinus* is mediated by an α-macroglobulin and possibly other thioester-containing proteins (Burešová et al., 2009; Urbanova et al., 2017). In *R. microplus*, phagocytic activity has been assessed, also demonstrating that plasmatocytes are the main cell type involved in yeast phagocytosis (Pereira et al., 2001). Moreover, it is already documented that ticks can phagocytize EPF (Figure 1), as demonstrated by Fiorotti et al. (2019; 2022) and Correa et al. (2021). Moreover, ultrastructural analyses in *R. microplus* revealed dose-dependent cytotoxicity of *Metarhizium robertsii* to hemocytes, indicating an arms race where fungal virulence factors damage immune cells while hemocytes attempt fungal clearance (Fiorotti et al., 2019). Unfortunately, in invertebrates, especially in ticks infected by EPF, the signaling pathways of phagocytosis are still poorly understood, as few studies have elucidated the molecular mechanisms that activate hemocytes.

In insects, hemocyte-mediated phagocytosis can be driven by complement-like proteins that opsonize various pathogens (Browne et al., 2013). Several molecules related to the mammalian complement system have already been described in ticks (Burešová et al., 2011; Urbanova et al., 2014). In recent studies, through RNAi silencing of tick thioester-containing proteins (TEPs), it was demonstrated that C3-like complement factors together with α2-macroglobulin protease inhibitors released into the hemolymph are key mediators of *M. robertsii* phagocytosis (Fiorotti et al., 2022). In immune responses against fungi, it has also been shown that CL5a and CL5b isoforms bind to these microorganisms, suggesting a unique role for these lectins in recognizing and differentiating these pathogens (Zhu et al., 2006).

Studies have shown convergence between autophagy and phagocytosis (Ferguson & Green, 2014). Autophagic machinery is also used to remove pathogens and direct them to lysosomal degradation, and is therefore considered a process of innate immune response against microbial infection (Mao & Klionsky, 2017). From this perspective, in invertebrates, organisms lacking acquired immunity, autophagy may play a fundamental role in protection against potential pathogens. Although the role of autophagy has been extensively investigated in model organisms such as *Drosophila melanogaster* and *Caenorhabditis elegans*, its function in other invertebrate groups remains poorly understood. Nevertheless, evidence suggests that autophagic processes may be activated in response to EPF (Malagoli et al., 2010; Lamiable et al., 2016; Fiorotti et al., 2019).

Unlike phagocytosis, nodule formation results in an overlaid sheath of multiple layers of hemocytes around the invader (Gillespie et al., 1997). Currently, there is no information in the literature elucidating this process in ticks challenged with EPF. Some invertebrate genetics studies have shown that melanization and nodulation responses share components and mechanisms, mainly involving activation of prophenoloxidase (PPO). Phenoloxidase (PO) combats infection by converting tyrosine into dopa, an important substrate that can be oxidized by PO into dopaquinone or decarboxylated by dopa decarboxylase (Ddc) into dopamine, resulting in melanin or sclerotin (Sideri et al., 2008). PO has also been reported to aid in melanization, phagocytosis, and nodulation of pathogens in several insect species (Cerenius et al., 2008), but little is known about these processes in ticks. Moreover, dopamine (DA) seems to modulate hemocyte activity in arthropods as demonstrated in *R. microplus*, where exogenous and pharmacologically modulated DA altered hemocyte phagocytosis and survival outcomes after *Metarhizium* challenge, demonstrating a neuro-immune axis influencing antifungal defense (Correa et al., 2021; Bório et al., 2022).

Melanization is an immediate immune response in arthropods, leading to the physical encapsulation of pathogens in a dense layer of melanin and the generation of toxic metabolites that can damage certain pathogens. This process has been visualized in insects and ticks infected by EPF, demonstrating melanization of fungal structures and even of the arthropod cuticle (Ment et al., 2012; Yassine et al., 2012).

#### Humoral immune response

The immune response via hemocytes is considered the first line of defense; however, during massive and prolonged infection, the cellular response may be depleted. Under these circumstances, the humoral immune response arises as a complement to the cellular response. In invertebrates, four main pathways are involved in activating the immune response: Toll, immune deficiency (IMD), Janus kinase (JAK/STAT), and Jun N-terminal kinase (JNK) (Capelli-Peixoto et al., 2017). The Toll pathway is activated by bacteria, viruses, and fungi; the immune deficiency pathway is activated only by Gram-negative bacteria; and the JAK/STAT pathway is similar to cytokine signaling in mammals (Shuai et al., 1993).

In ticks, most components of the Toll pathway have been described and found in the genome, indicating conservation of this pathway in these arthropods (Palmer & Jiggins, 2015; Bechsgaard et al., 2016; Gulia-Nuss et al., 2016). The presence of multiple Toll receptors and Spätzle cytokines suggests that the Toll pathway is also involved in regulating tick immune responses against invading microorganisms and pathogens transmitted to vertebrate hosts (Rosa et al., 2016).

The IMD pathway was first described in *D. melanogaster.* Ticks lack several key components of this pathway, such as transmembrane receptors and Imd (Severo et al., 2013; Gulia-Nuss et al., 2016; Rosa et al., 2016), yet the pathway can still function (Shaw et al., 2017). In mosquitoes, this pathway appears to play a very important role in antifungal responses, with transcription factors highly expressed when mosquitoes are challenged by EPF (Ramirez et al., 2018), although in ticks the mechanisms triggered upon challenge are still poorly understood.

The Janus Kinase (JAK-STAT) pathway was first described in mammals and plays a crucial role in antiviral responses (Dupuis et al., 2003; Ho et al., 2005). More recently, this pathway was identified as not belonging strictly to insect humoral immunity; its role in immunity is due to links with the Toll and IMD pathways (Myllymäki et al., 2014). In ticks, this pathway is also conserved compared with *Drosophila* (Palmer & Jiggins, 2015; Bechsgaard et al., 2016), except for *upd* gene (Rosa et al., 2016).

In invertebrates, various compounds in the hemolymph have proven active in recognizing and eliminating pathogens; antimicrobial peptides therefore play a very important role in responses against parasites, fungi, and bacteria (Bulet et al., 2004). Most antimicrobial peptides (AMPs) are cationic and amphipathic, capable of forming transmembrane channels, and are part of the innate immune response of many animal species, including vertebrates and invertebrates (Ludtke et al., 1996; Yang et al., 2017). Currently, studies involving AMPs in ticks are scarce; the only ones described in these arthropods are defensins, hemocidins, ixodidins, lysozymes, and microplusins (Fogaça et al., 2004; Fogaça et al., 2006; Sonenshine & Hynes, 2008; Belmonte et al., 2012).

In ticks, defensins have also been detected in hemocytes (Johns et al., 2001; Fogaça et al., 2006), ovary (Lima et al., 2006), salivary glands (Lima et al., 2006), and gut (Kopácek et al., 1999), with activity against Gram-negative and Gram-positive bacteria, fungi, and protozoa (Wang & Zhu, 2011; Couto et al., 2018). Hemocidins are hemoglobin fragments demonstrated in several animal species. In addition to being reported in vitro, they have also been identified in the gut of the tick *R. microplus* and can be active against Gram-positive bacteria and fungi (Sforça et al., 2005; Belmonte et al., 2012). Despite these findings, little is known about the specific contribution of AMPs during tick infections with EPF, highlighting an important gap for future investigations.

#### Redox metabolism in ticks

Oxidative stress can be simply defined as an imbalance between oxidants and antioxidants leading to disruption of redox signaling and control and/or molecular damage (Sies et al., 2017). In ticks, heme and iron homeostasis plays a central role in development and reproduction in several species (Galay et al., 2014; Hajdušek et al., 2016). Moreover, the toxicity of reactive oxygen species plays a significant role in arthropod immune responses. The ProPO cascade leads to melanization of fungal structures either on the cuticle or even within capsules formed by hemocytes (Butt et al., 2016), and its activation is mediated by β-1,3-glucans of the fungal cell wall and by damaged host tissues (Cerenius et al., 2008). Products of melanization, such as reactive oxygen species, destroy pathogens but are also toxic to hosts (Nappi & Christensen, 2005). However, studies of oxidative stress in tick hemocytes are scarce (Pereira et al., 2001), since most research focuses on embryonic cells (Kalil et al., 2017), and little is known about the effect of EPF virulence on ROS generation in a host.

In *Galleria mellonella,* ROS production in the hemolymph increases significantly after infection by *Cordyceps* sp. but not by some *Metarhizium* species, suggesting that this increase may be related to necrotic death of hemocytes (Kryukov et al., 2020). However, ROS production increases significantly in *Cordyceps* infection despite a drop in phenoloxidase activity, demonstrating that other routes or mechanisms of ROS production may also be involved (Kryukov et al., 2020). In *Metarhizium* sp. infection, there is a tendency toward decreased ROS production, consistent with reduced PO activity in the hemolymph (Kryukov et al., 2020). Some authors thus suggest that upregulation of SOD and catalase alters host redox regulation during infection. ROS production may also be related to fungal virulence, as increased ROS is observed after infection by less virulent fungi (Vorontsova et al., 2018; Kryukov et al., 2020).

Although the tick immune system lacks some canonical components described in insects, it has evolved unique and effective strategies to counter EPF. Cellular responses mediated by hemocytes, together with humoral pathways such as complement-like proteins, antimicrobial peptides, and redox metabolism, provide an integrated defense that can limit fungal development. Accordingly, recent advances demonstrate that ticks combine classical immune mechanisms with species-specific adaptations, highlighting a complex and multifactorial antifungal immunity. Understanding these processes not only clarifies host–pathogen interactions but also supports the development of fungal-based biocontrol strategies to overcome tick immune defenses.

## Microbiome-Mediated Interactions

The tick microbiome refers to the community of commensal, symbiotic, and pathogenic microorganisms that inhabit different areas of the tick body (Narasimhan & Fikrig, 2015). It is recognized as a complex and dynamic system that interacts intensely within a macrosystem that includes the host organism, and in the case of ticks, it is composed of microorganisms found not only in the host’s blood, but also on the skin and in the surrounding environment. This microbial community is fundamental to diverse aspects of tick biology, influencing their physiology, environmental adaptation, and vector competence (Narasimhan et al., 2014; Bonnet et al., 2017; Kitsou et al., 2021; Pavanelo et al., 2020). As an example, bacteria of the genera *Coxiella* and *Francisella* provide crucial nutritional supplementation, synthesizing B vitamins and cofactors that are necessary for survival and reproduction due to the constraints of the blood-feeding lifestyle. These alliances also support tick metabolism, including nitrogen recycling and osmoregulation, and are indispensable for successful reproductive output (Narasimhan & Fikrig, 2015; An et al., 2022; Noguerales et al., 2025). Experimental elimination of symbionts, for instance using antibiotics against *Coxiella* in *Rhipicephalus sanguineus*, can irreversibly impair nymphal development, adult engorgement weight, and egg hatching rates (An et al., 2022). Similarly, *Coxiella* endosymbiont from *R*. *microplus* proved to be essential for the tick to reach the adult life stage, as under antibiotic treatment no tick was able to progress beyond the metanymph stage (Guizzo et al., 2017). Despite all this knowledge, many authors agree that the current understanding of microbial community variation across tick species, life stages, and sex and the underlying processes remains limited (Aguilar-Díaz et al., 2021; Noguerales et al., 2025).

In terms of vector competence, some studies reported that the microbiota may impact the tick’s ability to transmit pathogens, modulating the acquisition, colonization, and dissemination of tick-borne agents (Aguilar-Díaz et al., 2021; Noguerales et al., 2025). Because ticks are hematophagous, the midgut is a pivotal tissue, serving as the primary site of pathogen entry and initial colonization. Manipulation of the gut microbiota, whether through external intervention or naturally through the pathogens themselves, directly affects the presence of vertebrate pathogens in the tick gut (Narasimhan et al., 2014; Mesquita et al., 2023), most likely altering the tick's vectorial capacity. For example, altering the microbiota in *Ixodes scapularis* can compromise the integrity of the peritrophic matrix, subsequently impairing the colonization of *Borrelia burgdorferi* (Narasimhan et al., 2014). Conversely, some pathogens, such as *Anaplasma* sp., have evolved mechanisms that may enhance tick survival, including increasing cold tolerance (Neelakanta et al., 2010) highlighting a complex coevolutionary relationship that benefits the vector.

Since many factors (such as host characteristics and environmental conditions like temperature, humidity, and geographic location) can influence the composition of the tick microbiome (Gurfield et al., 2017; Brinkerhoff et al., 2020), it is not surprising that biotic stressors also play an important role. Among these, pathogen presence or infection status can lead to significant microbiome modulation, resulting in pathogen-induced dysbiosis or the dominance of specific bacterial taxa. Within this context, it becomes crucial to understand how this tripartite system (comprising the tick, entomopathogenic fungi, and disease-borne microorganisms) responds to additional external agents.

In a recent study, Mesquita et al. (2023) aimed to explore how the initial steps of entomopathogenic fungal infection (topical treatment) may trigger changes in the gut bacterial community of *R. microplus*, and whether disrupting the pre-existing gut bacterial community (using an antibiotic) affects the tick's susceptibility to the fungus. The study reported that challenging *R. microplus* with *M. anisopliae* changes the tick gut bacterial community. The combined use of tetracycline (through ingestion) and the fungus (topical treatment) altered the bacterial community by increasing its diversity. Crucially, the susceptibility of the tick to the entomopathogenic fungus remains unaffected by previous antibiotic therapy. Additionally, in Mesquita et al. (2023) the authors highlighted that the genus *Ehrlichia* was found in the guts from untreated tick females, but it was not detected in the fungus-treated groups. These findings suggest that fungal treatment may influence the dynamics of pathogens relevant to vertebrate hosts. However, to confirm this potential effect, specific studies addressing vector competence and pathogen transmissibility would be required.

## Formulation Technologies and Delivery Systems

The success of biocontrol agents in agriculture and livestock depends not only on their inherent efficacy, but also on how they are formulated and delivered in the field. Unlike chemical pesticides, biocontrol products based on EPF are composed of living organisms or biologically derived molecules that are highly sensitive to environmental stress, including ultraviolet radiation, desiccation, temperature, and pH (Burges & Jones, 1998; Jaronski, 2010). In this way, formulation technologies are critical to maintain viability, prolong shelf life, and optimize the delivery to the target environment. For agricultural purposes, a reasoned approach includes the propagule type (spore, conidium, blastospore, microsclerotium), exposure route (foliar, soil/rhizosphere, seed); field microclimate (UV, temperature, relative humidity); product concentration and delivery device. Likewise, in livestock, effective strategies for controlling ticks must match the biological control agent, the ectoparasite species, the route of exposure (topical, environmental), and the formulation and application system suited to animal health practices. In the following sections, particularly the main approaches related to fungal-based treatment strategies for tick control, are discussed in detail.

EPF such as *M. anisopliae*, *B. bassiana*, and *C. fumosorosea* are among the most studied and widely applied microbial control agents in agriculture. More recently, researchers have also advanced with the use of species such as *C. javanica* and *Purpureocillium lilacinum* (Angelo et al., 2012; Pereira et al., 2024; Li et al., 2025; Moura et al., 2025). Their success is strongly influenced by the biological characteristics of their propagules, which define both their ecological performance, and the formulation strategies required for commercialization. Unlike bacterial endospores, which are extremely resistant, fungal propagules are relatively sensitive to ultraviolet radiation, desiccation, and temperature, making formulation a decisive step for ensuring field persistence and efficacy (Inglis et al., 2001; Jaronski, 2010).

The most widely used propagules in EPF-based products are aerial conidia. These structures are hydrophobic, conferring some tolerance to desiccation and enhancing adhesion to insect cuticles (Inglis et al., 2001; Birnbaum et al., 2021). However, their hydrophobicity limits dispersibility in water, often requiring surfactants or dispersants for suspension concentrates (Fadiji et al., 2024). Oil-based formulations have shown strong advantages for aerial conidia, since vegetable or mineral oils improve deposition on insect cuticles, reduce evaporative stress, and maintain efficacy under low relative humidity conditions (Umaru & Simarani, 2022; Mahot et al., 2025). The addition of stickers and spreaders further increases rainfastness, while UV protectants such as lignin, carbon black, or titanium dioxide are widely used to mitigate photoinactivation during foliar applications (Kaiser et al., 2018). In dry formulations such as wettable powders (WP) or water-dispersible granules (WDG), conidia can retain viability if water activity is tightly controlled (Burges, 2012; Lacey, 2012; Lei et al., 2022), whereas in aqueous formulations they lose infectivity more rapidly unless stabilized with humectants and antioxidants (Seabra et al., 2024).

In addition to aerial conidia, blastospores are increasingly considered as alternative propagules. Their production via liquid fermentation is more economical and scalable than solid-state methods, making them attractive for industrial mass production. However, their fragility during drying and storage processes remains a major issue: blastospores are especially vulnerable to desiccation, UV exposure, and thermal stress, which severely limit their shelf life unless advanced stabilization strategies are developed. For example, cultivation in nitrogen-rich media has been shown to enhance desiccation tolerance of blastospores (Mascarin et al., 2024), and efforts to improve desiccation tolerance have been proposed to mitigate their short shelf stability (Dietsch et al., 2021). Suspension concentrates enriched with protective solutes such as trehalose, skim milk, or polyvinyl alcohol coating can extend their viability, and microencapsulation in alginate or starch matrices can also demonstrate positive effects on stability (Hermann et al., 2023).

Another promising fungal propagule for formulation is microsclerotium. These are compact, melanized hyphal aggregates capable of surviving extended periods of desiccation and regenerating conidia when conditions become favorable. Microsclerotia are more tolerant to environmental stress than conidia or blastospores and thus are especially suited for soil application (Paixão et al., 2021). When incorporated into granules or pellets, they germinate *in situ*, producing infective conidia that persist in the soil and provide long-term pest suppression. Although the onset of insect mortality is slower compared to direct application of conidia or blastospores, microsclerotia act as a reservoir for sustained production of infective units and reduce the frequency of applications (Rivas-Franco et al., 2020).

### Excipients

The selection of excipients is a cornerstone in the development of microbial biocontrol formulations, as these substances not only ensure physical stability but also preserve the viability and infectivity of propagules during storage, handling, and field application. Carriers, oils, adjuvants, dispersants, humectants, UV protectants, antioxidants, encapsulating matrices, and preservatives are combined in different ways to meet the specific requirements of each microbial agent and its target environment in agriculture.

Mineral carriers such as talc, kaolin, diatomite, and bentonite are widely used in solid formulations, where they provide bulk, flowability, and moisture buffering capacity in WP and WDG. These carriers reduce aggregation, improve handling, and facilitate dispersion in spray tanks (Burges, 2012). Organic carriers, including starch, maltodextrin, microcrystalline cellulose, and lignin derivatives, are important in spray-drying and encapsulation processes, where they protect spores and conidia against thermal and osmotic shocks and promote uniform dispersion in aqueous suspensions (Mishra et al., 2015).

Vegetable and mineral oils, as well as oil-based adjuvants, are especially advantageous for hydrophobic conidia of EPF such as *Metarhizium* sp. and *Beauveria* sp. (Wraight et al., 2016; De Oliveira et al., 2018). In oil dispersions (OD) and emulsifiable concentrates (EC), emulsifier packages, typically blends of non-ionic surfactants with carefully adjusted hydrophilic–lipophilic balance (HLB), are required to ensure tank mix compatibility and physical stability during application.

In aqueous formulations, dispersants and stickers play a critical role. Lignosulfonates and polyacrylates prevent sedimentation in suspension concentrates (SC), while non-ionic surfactants improve foliar coverage. Latex and rosin ester stickers enhance adhesion and rainfastness, which is essential for the performance of fungi exposed to precipitation in field environments (Fravel, 2005; Glare et al., 2012).

Humectants and osmoprotectants, including glycerol, sorbitol, polyethylene glycol, trehalose, and skim milk powder, mitigate desiccation stress and improve tolerance to drying, both during industrial processing and after field application. Their protective effects are particularly important for blastospores, which are more fragile than aerial conidia. The addition of trehalose or milk solids has been shown to enhance post-processing viability and extend shelf life in aqueous formulations (Wraight et al., 2001; John et al., 2011).

Protection against UV and oxidative stress is another fundamental requirement, particularly for fungal conidia applied to foliage. Additives such as lignin, titanium dioxide, carbon black, and optical brighteners reduce photoinactivation, thereby extending persistence on plant surfaces (Burges, 2012).

In recent years, encapsulation and controlled-release systems have emerged as advanced strategies to improve microbial survival and performance. Natural polymers such as alginate, pectin, carrageenan, chitosan, and starch create protective matrices that regulate hydration, and provide controlled release of microbial propagules in the soil. Encapsulation also enables the co-delivery of micronutrients and protective agents, which can enhance microbial establishment in the target habitat (Celestino & Oliveira, 2022; Riseh et al., 2022; Colin et al., 2024).

Another critical aspect in formulation design is the control of contamination, pH, and water activity. Preservative agents such as potassium sorbate and sodium benzoate help suppress contaminants without affecting the target microorganism (Islam et al., 2024).

### Delivery systems in livestock

Among the various methods used for tick control, the direct treatment of host animals (particularly via spraying and dipping vats) remains the predominant and most extensively implemented approach (Abbas et al., 2014; Klafke et al., 2024).

Although dipping vats are theoretically effective for tick control, their practical application has several limitations. According to Klafke et al. (2024), common issues include the accumulation of organic debris at the bottom of the vat, the use of off label acaricides, inadequate mixing of the active ingredient, incorrect replenishment procedures, and water infiltration, all of which contribute significantly to treatment failure. The high volume of formulation required, and the cost will make this method less viable for fungal applications. The pour-on method, although practical and fast to apply, is not well-suited for EPF-based products. Unlike synthetic acaricides (whose active ingredients can diffuse efficiently across most of the animal’s skin surface) EPF require a sufficiently high number of viable propagules to reach the tick and initiate infection. In pour-on systems, the limiting factor is not necessarily incomplete body coverage, which can occur effectively through the natural spread of the formulation along hair and skin secretions, but rather the extremely low dose applied. To remain effective, the formulation would need to contain very high fungal concentrations while still being able to disperse and remain viable during dilution across the animal’s skin.

Spraying stands out as a practical and suitable delivery method for synthetic acaricides (Klafke et al., 2024) and it is also more suitable for EPF as it allows a broad and uniform coverage of the animal’s body, ensuring better contact between the fungal spores and the ticks - which supports the mode of action of these microbial agents. Spraying can be performed using manual or power sprayers, and even using cattle spray racers (Moraes et al., 2023; Barbieri et al., 2023), and for EPF, its effectiveness is enhanced when applied during periods of low UV radiation, with attention to nozzle calibration, spray pressure, and direction against the hair coat to maximize deposition on tick-prone areas (Bahiense et al., 2008; Xu et al., 2023; UDAF, 2020).

Despite its advantages, spraying has its limitations. It often requires individual animal handling, which can be labor-intensive and impractical for large herds (Moraes et al., 2023). Cattle spray races, or automated walk-through spraying systems, present a scalable alternative by increasing animal throughput and reducing labor requirements. Notably, their use and effectiveness have been demonstrated with oil-based formulations of *M. anisopliae*, supporting their potential use in fungal acaricide delivery (Barbieri et al., 2023). It can be an interesting alternative indicated for big farmers with large cattle herds to increase their adhesion to biological control, since it allows a quick way to treat hundreds of animals (Barbieri et al., 2023). On the other hand, these systems may involve an initial cost for purchase and facility installation, and the need for continued maintenance (Moraes et al., 2023).

Given these advantages and challenges, an integrated approach that combines on-animal and environmental applications is promising for the future of tick control with EPF. It's important to recognize that tick control is primarily an environmental issue, as a significant portion of the tick life cycle occurs off host. Therefore, innovation in delivery technologies and strategies is crucial. While direct application remains the most practical, economically viable, and widely accepted method (especially considering that many cattle producers already use spraying equipment for chemical acaricides) there is a need to break paradigms and explore environmental applications. This includes the use of self-propelled sprayers, boom sprayers, and drones for pasture treatment. However, more field-based studies are needed to validate these approaches and optimize their use for effective tick control.

The effectiveness of microbial control agents in agriculture depends not only on their inherent virulence but also on the delivery method used in the field. Foliar applications are typically performed with hydraulic booms, air-blast sprayers, or mist blowers, where droplet size and low-shear pumping are key to maintaining propagule viability (Jiao et al., 2025). Electrostatic spraying improves deposition on complex canopies with lower carrier volumes (Herkins et al., 2025). Unmanned aerial systems (UAS/drones) have gained attention for hotspot applications, but their performance depends on stable, low-foaming formulations and careful droplet management (Faers et al., 2024). Recent studies with ticks emphasize matching formulation type with application tools, especially for dry powder systems (Pereira et al., 2024). Additionally, drone-based spraying is gradually being explored for precision livestock and hotspot management (Pereira et al., 2024; Andrade et al., 2025).

For soil and rhizosphere applications, in-furrow liquids or granules deliver propagules directly to moist zones favorable for survival. Drip irrigation and chemigation can also distribute microbial formulations, provided filter maintenance prevents clogging. Banding and side-dressing place microbes near roots, enhancing persistence (Boari et al., 2008; Bejarano & Puopolo, 2021).

Seed delivery through film-coating or pelleting ensures accurate dosing and good adhesion with polymer binders (Rocha et al., 2019). Additionally, “attract-and-infect” devices using semiochemicals or pheromones improve pest–pathogen contact and protect microbes from UV and desiccation, increasing field persistence (Glare et al., 2012; Dembilio et al., 2018; Hassemer et al., 2020).

This section presented the main application tools used in livestock for tick control, as well as common agricultural technologies that can be adapted for the application of EPF in the environment, targeting the non-parasitic phase of ticks. This approach aims to encourage reflection and foster innovation, aligning with best practices in parasite control, especially considering that management must also be environmental, since most of ticks are found off host. The evolution of delivery systems reveals promising opportunities by integrating technologies across agricultural and livestock sectors. The convergence of tools used by livestock and crop producers, often represented by the same individual, allows for resource optimization, avoiding the need for new equipment and promoting operational synergies. Integrated pest management, applied to both agriculture and livestock, gains strength through the possibility of joint application of compatible microorganisms targeting multiple pests, such as nematodes, spittlebugs, phytopathogenic fungi, and ticks. This practice not only reduces costs but also enhances the sustainability and efficiency of production systems, pointing to a trend of innovation and best practices in the control of parasites of veterinary and agricultural importance.

Ultimately, this integration underlines the practicality and accessibility of biological delivery systems for producers who are both farmers and cattle raisers. Shared spray assets, drones, formulations that suit both crop and pasture environments, and the reduced need for redundant infrastructure all lower entry barriers. These factors help drive adoption of biologically based control methods, improving sustainability, cost-efficiency, and innovation in mixed production systems.

## Integration with Chemical Acaricides

The integration of tick control methods has been proposed as a more sustainable alternative, combining the rational use of synthetic, biological, and immunological acaricides with genetic improvement and environmental management (Mollong et al., 2025).

The combination of biological and synthetic pest control is already widely used in agricultural crops in Brazil, as demonstrated in a recent study by Medyk et al. (2025), who evaluated the productivity of a corn (*Zea mays*) plantation using different pest control treatments. The treatments tested included synthetic insecticides (Imidacloprid combined with Beta-cyfluthrin), a biological insecticide (*B. bassiana*), and a combination of both. According to their results, treatments with synthetic insecticides and the combination of synthetic and biological insecticides significantly increased productivity, as assessed by the parameters of number of grains per ear and thousand-grain weight, compared to the biological treatment alone and the control group. The data demonstrated that, to optimize pest control and ensure higher productivity, the combination of chemical and biological methods is the most effective strategy. These results emphasize the importance of integrated pest management practices to achieve efficient and sustainable agricultural production.

In the context of integrated tick control, using EPF in combination with synthetic acaricides has proven to be a promising strategy (Webster et al., 2015), as it is effective even in tick populations that are already resistant to synthetic acaricides (Bahiense et al., 2006; Barci et al., 2009; Webster et al., 2015; Carneiro et al., 2022). However, studies have demonstrated that interactions with certain synthetic acaricides may negatively affect fungal germination and colony development (Soares & Monteiro, 2011; Carneiro et al., 2022). These findings reinforce that the compatibility between the control agents is highly context-dependent and is influenced by factors such as the fungal species, isolate, the type of propagule, the active ingredient, as well as the formulation and adjuvants employed (Barci et al., 2009; Alonso-Díaz & Fernández-Salas, 2021).

One approach to assessing fungus-acaricide interactions is through *in vitro* assays, in which culture media are supplemented with defined concentrations of acaricides and subsequently inoculated with fungal suspensions. Barci et al. (2009) evaluated the compatibility between two *B. bassiana* isolates (IBCB66 and IBCB21) with flumethrin combined with coumaphos, deltamethrin, dichlorvos combined with cypermethrin, dichlorvos combined with chlorpyrifos, cypermethrin high cis, dichlorvos combined with cypermethrin high cis, cypermethrin, and amitraz used to control *R. microplus*. The authors observed an isolate-dependent variation in compatibility, with IBCB66 exhibiting higher tolerance to the acaricides deltamethrin, high-cis cypermethrin, and amitraz, as these compounds did not significantly impair key fungal developmental parameters, including vegetative growth, conidial production, and viability. In contrast, IBCB21 exhibited lower tolerance to most of these acaricides. In that study, among the products tested, only deltamethrin exhibited no apparent toxic effects on either isolate. Similarly, but using *M. anisopliae* ESALQ E9, Soares & Monteiro (2011) evaluated the compatibility of the fungus with amitraz combined with chlorpyrifos, cypermethrin, dichlorvos combined with chlorfenvinphos, cypermethrin combined with chlorpyrifos and citronellal, spinosad, in different concentrations. Most of the products tested were not compatible with this *M. anisopliae* isolate, as they inhibited fungal growth and sporulation. Only the acaricides formulated with cypermethrin and spinosad exhibited some compatibility for use in a possible associated use strategy. At this point, it is reasonable to mention that the acaricide’s adjuvants in the commercial formulations may also be as important as drugs themselves to determine their compatibility with fungal agents.

In addition to these tests in culture media, some studies have evaluated the association of EPF and acaricides on ticks. In the study conducted by Bahiense et al. (2006), the authors evaluated the compatibility between the fungus *M. anisopliae* ESALQ 959 and a synthetic acaricide based on deltamethrin against a strain of *R. microplus* resistant to this pyrethroid. The authors observed that the combination of pyrethroid and fungus resulted in higher mortality rates of *R. microplus* larvae than those obtained with the respective uncombined concentrations, demonstrating that combining the two methods improved efficacy.

The effectiveness of combining EPF with chemical acaricides has also been demonstrated in field trials for controlling *R. microplus*. Webster et al. (2015) combined cypermethrin and chlorpyrifos with *M. anisopliae* TIS-BR03 and evaluated its effectiveness in field tests on the Jaguar strain of *R. microplus* (multiresistant to six classes of chemical acaricides). Four experimental groups were formed, which were sprayed with G1: acaricide solution; G2: fungal suspension alone; G3: combination of fungal suspension with acaricide solution; G4: control group that received no treatment. As a result, the authors observed efficacy values of 71%, 56%, and 97% for groups G1, G2, and G3, respectively, demonstrating that the combination of the two methods (biological and chemical) increased the efficacy over the tick population, suggesting that this could be a tool for controlling resistant strains. Additionally, Webster et al. (2015) proposed the integrated use of acaricides in combination with fungal agents as a recommendation that may help to popularize biological control among cattle ranchers, especially for those still using only traditional practices.

Given these promising results obtained using EPF in both in vitro tests and field experiments, research is being conducted to improve the production and formulation of these microbial agents. Accordingly, different types of fungal propagules can be used, such as aerial conidia (Bahiense et al., 2006), microsclerotia (Mascarin et al., 2014; Mascarin et al., 2019; Marciano et al., 2021), and blastospores (Bernardo et al., 2018; Corval et al., 2021). These propagules were recently tested by Carneiro et al. (2022), who evaluated their compatibility with different chemical acaricides. For aerial conidia, *M. robertsii* ARSEF 2575 and *Metarhizium pingshaense* LCM S09 were used, along with acaricides based on amitraz, deltamethrin, and a commercial combination of cypermethrin + chlorpyrifos + citronellal. The conidial suspensions were kept in contact with each of the acaricides for 1, 5, 10, and 24 hours and were subsequently inoculated into culture medium for quantification of colony-forming units. For the compatibility test of blastospores and microsclerotia with acaricides, the same methodology described above for conidia was used. However, for microsclerotia, after incubation with synthetic acaricides, an aliquot of this propagule was transferred to culture medium, and germination was evaluated after 72 h. In addition to these tests in culture medium, the authors also evaluated the effect of the association of deltamethrin with the different propagules of *M. robertsii* or *M. pingshaense* on engorged females of *R. microplus*. In general, the conidia and microsclerotia propagules of the fungal isolates tested were more compatible with synthetic acaricides than the blastospores. In bioassays with *R. microplus* engorged females, the combination of *M. pingshaense* and deltamethrin achieved the highest level of control compared with either the fungus or the synthetic acaricide applied alone (Carneiro et al., 2022).

Most studies evaluating the association between EPF and synthetic acaricides were conducted using the microorganism in aqueous suspension (Bahiense et al., 2006; Webster et al., 2015; Carneiro et al., 2022). Although this method of application is practical and widely used in laboratory and field experiments, it exposes fungal propagules to various abiotic factors, in addition to possible adverse chemical interactions with the active ingredients and adjuvants present in acaricide formulations. However, when microorganisms are formulated with protective agents, a significant improvement in the compatibility and stability of fungi when combined with chemicals can be observed, as demonstrated by Lopes et al. (2011). These authors evaluated the protective capacity of an oily formulation (dispersion oil) for conidia of *M. anisopliae* (ESALQ 1037) and *B. bassiana* (CG 1027), associated with the chemical fungicides triadimefon and carbendazim. The results showed that the effect of the fungicides was nil or minimal for EPF conidia, as there was no reduction in fungal germination rate or virulence in the groups containing the oil formulation when compared to the aqueous group. Compounds such as vegetable oils, emulsifiers, neutral surfactants, and inert carriers act as physical barriers, reducing direct contact between fungal conidia and the active ingredients in acaricides. These additives help minimize the harmful effects of solvents, stabilizers, and surfactants (Kinyanjui et al., 2025).

These studies reinforce the importance of integrating the different control tools available for ticks. From the literature, combining synthetic acaricides with entomopathogenic fungi may be feasible. However, all the factors discussed above must be considered, as several determinants can negatively influence the interaction between fungi and chemicals. It is important to conduct further studies evaluating the application forms and combinations, but above all, more tests under field conditions.

## Field Applications and Real-World Efficacy

**Figure 2:**
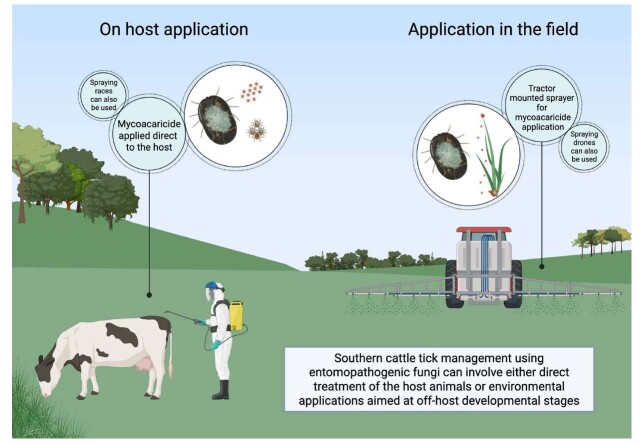
Overview of cattle tick control strategies using entomopathogenic fungi, including direct application to infested animals during the parasitic phase and field application targeting non-parasitic tick stages.

As mentioned previously, tick control using fungi can rely on two main approaches: (i) the application of EPF directly onto infested animals, targeting tick control (larvae, nymphs, and adults) during their parasitic phase; or (ii) the application of these fungi to the soil, targeting tick control during their non-parasitic phase (for monoxenous ticks: engorged females, eggs, and unfed larvae; for heteroxenous ticks: eggs; larvae, nymphs and adults - unfed and engorged). Figure 2 exemplifies these different treatment approaches in the context of *R. microplus*.

### On-host application

To date, approximately 20 studies had been conducted in which the fungus was sprayed directly to the host, and varying levels of efficacy were observed (Table 1). The first field study involving direct application to animals was conducted using *B. bassiana* (1 × 10^8^ conidia/mL), which was applied to the ears of cattle infested with *Dermacentor nitens*. After 35 days, the treatment achieved 70% efficacy. However, the fungus did not affect the reproductive biology of the engorged females recovered from the animals (Monteiro et al., 2003). The first studies with *R. microplu*s were conducted using *M. anisopliae* s.l. applied to cattle artificially infested under stall test (Bahiense et al., 2007) and to naturally infested cattle under field trials (Alonso-Díaz et al., 2007). In the study conducted by Bahiense et al. (2007), an aqueous suspension of *M. anisopliae* (ESALQ-959) at a concentration of 8 × 10^8^ conidia/mL was used, resulting in an average mortality rate of 33% among ticks recovered from animals and a 19% reduction in the oviposition index. Alonso-Díaz et al. (2007) performed biweekly applications of a *M. anisopliae* (Ma34) suspension at 1 × 10^8^ conidia/mL for two months and observed, from the second application onward, a significant reduction in tick infestation, with efficacy ranging from 40% to 91% over the experimental period.

**Table 1:** Studies on the use of entomopathogenic fungi for tick control via host or environmental application.

**Site of application**	**Reference**	**Type of study**	Fungi × Tick*	**Strain / Suspension**	**Results**
**On the host**	Monteiro et al. (2003)	Spraying (1×) on stabled cattle artificially infested	Conidia of *B. bassiana* s.l. × *D. nitens* (L, N and A)	Strain ESALQ 986 (8×10^8^); aqueous suspension (Tween 80 0,1%)	Efficacy of 70.1% 35 days after the treatment. However, the fungus did not affect the reproductive biology of the engorged females recovered from the cattle.
	Bahiense et al. (2007)	Spraying (1×) on stabled cattle artificially infested	Conidia of *M. anisopliae* s.l. × *R. microplus* (L, N and A)	Strain ESALQ 959 (8×10^8^); aqueous suspension (Tween 80 0,1%)	Reported tick mortality of 33% and reduction in the egg production index of engorged females.
	Alonso-Díaz et al. (2007)	Spraying (1× every 15 days for 2 months) on naturally infested cattle	Conidia of *M. anisopliae* s.l. × *R. microplus* (L, N and A)	Strain Ma34 (1×10^8^); aqueous suspension (Tween 80 0,1%)	From the second application onward a significant reduction in infestation was observed, with efficacy ranging from 40% to 91.2%.
	Bahiense et al. (2008)	Spraying (1×) on stabled cattle artificially infested	Conidia of *M. anisopliae* s.l. combined or not with deltamethrin × *R. microplus* (L, N and A)	Strain ESALQ 959 (1×10^8^); aqueous suspension (Tween 80 0,1%); deltamethrin (25 ppm)	No significant difference in tick mortality was observed between the group treated with deltamethrin (38.6%) and the group treated with deltamethrin combined with the fungus (30.9%).
	Leemon et al. (2008)	Spraying (1×) on stabled cattle artificially infested	Conidia of *M. anisopliae* s.l. × *R. microplus* (L, N and A)	Strain ARIM16 (2 e 2,5 × 10^8^), oil-based suspension (10% vegetable oil)	Ticks collected immediately after treatment and incubated in the laboratory at 28 °C exhibited 100% mortality within 2 days.
	Souza et al. (2009).	Spraying (1×) on stabled horses artificially infested	Conidia of *B. bassiana* s.l. × *D. nitens* (L, N and A)	Strain ESALQ 986 (1 × 10^9^), cellulose polymerized gel	Reduction in tick counts, with efficacy ranging from 18% to 80% over a 28-day evaluation period.
	López et al. (2009)	Spraying (3× at 7-day intervals) on naturally infested cattle	Conidia of *M. anisopliae* s.l. × *R. microplus* (L, N and A)	Strain 137bm (1×10^8^); formulation containing lactose, talc, and xanthan gum (concentration not reported)	A 75% reduction in adult tick infestation (> 4 mm) was observed from the third week onward, along with reduced fecundity in engorged females recovered from treated animals.
	Kaaya et al. (2011)	Spraying (1× every 3 or 4 weeks for 12 months) on artificially and naturally infested cattle	Conidia of *M. anisopliae* s.l. × *R. evertsi* e *R. decoloratus* (L, N and A)	Strain NA1 (1 × 10^8^); oil-based suspension (20% vegetable oil) and Triton X-100 (0,05%)	The treatment resulted in reductions in tick counts of 83%, 79%, and 72% at three, four, and seven months post-treatment, respectively, while ticks collected from treated animals exhibited 93% mortality.
	Sousa et al. (2011)	Spraying (1×) on stabled cattle artificially infested	Conidia of *B. bassiana* s.l. combined or not *M. azedarach* x *R. microplus* (L, N and A)	(2,4 × 10^8^), combined or not with plant extract. aqueous suspension (Tween 80 0,1%)	Treatment with the fungus alone or in combination with the plant extract resulted in efficacies of 9% and 39%, respectively.
	Romo-Martínez et al. (2013)	Spraying (1×) on stabled cattle artificially infested	Conidia of *M. anisopliae* combined or not with *S. verbascifolium* x *R. microplus* (L, N and A)	Strain Ma379 (1 × 10^8^); *S. verbascifolium* (20.000 ppm)	The treatments reduced tick counts at 7, 14, and 21 days post-application, and recovered females exhibited reduced fecundity.
	Rot et al. (2013)	Spraying on artificially infested gerbils and rabbits	Conidia of *M. brunneum* × *R. sanguineus* (L, N and A)	Strain M-7; aqueous suspension (Triton X-100 0,01%), or oil-based suspension (10% mineral oil), or formulated in a starch–sucrose mixture; or in skim milk	Reduction in the number of unfed larvae and nymphs that developed and molted into nymphs and adults, respectively
	Camargo et al. (2014)	Spraying (1× every 15 days for 2 months) on stabled cattle artificially infested	Conidia of *M. anisopliae* × *R. microplus* (L, N and A)	Strain ESALQ 1037 and ESALQ E9 (1 × 10^8^); commercial product (Metarril^®)^; oil-based suspension (10% mineral oil)	Efficacy of 47.74% compared to the water control group and 40.89% compared to the oil control group.
	Webster et al. (2015)	Spraying (1× every 21 or 28 days for 161 days) on naturally infested cattle	Conidia of *M. anisopliae* combined or not with cypermethrin + chlorpyriphos × *R. microplus* (L, N and A)	Strain TIS-BR03 (1 × 10^8^); aqueous suspension (Triton X-100 0,02%); cypermethrin (0.02%) + chlorpyriphos (0.05%)	Efficacy of 71.1% with chemical acaricide treatment alone, 56.3% with *M. anisopliae* alone, and 97.9% with the combination of *M. anisopliae* and the chemical acaricide.
	Murigu et al. (2016a)	Spraying (1× every 7 days for 28 days) on naturally infested cattle	Conidia of *M. anisopliae* combined or not with Amitraz × *R. decoloratus* (L, N and A)	Strain ICIPE 07 (1 × 10^8^); oil-based suspension (15% vegetable oil); amitraz	Treatments with the fungus and with the fungus + amitraz combination resulted in a reduction in the number of ticks on cattle.
	Camargo et al. (2016)	Spraying (2× on day 0 and day +3) on naturally infested cattle	Conidia of *M. anisopliae* × *R. microplus* (L, N and A)	Strain ESALQ 1037 and ESALQ E9 (1 × 10^8^); commercial product (Metarril^®)^; oil-based suspension (10% mineral oil)	Average efficacy of 75.09% compared to the water control group and 46.59% compared to the oil control group.
	Zeina et al. (2022)	Spraying (2× on day 0 and day +2) on partially confined and artificially infested cattle	Conidia of *B. bassiana* combined or not with adjuvants × *R. microplus* (L, N and A)	Strain Bb-27, Bb-41 and Eco-Bb® (1×10^8^); Ballista® or Break-thru® (0,025%) with oil-based suspension (10% vegetable)	Tick mortality in treated groups ranged from 13% to 38%. All treatments reduced both the quantity and viability of eggs produced by engorged females recovered from the cattle.
					
	Alonso-Díaz et al. (2022)	Spraying (5× – days 0, 15, 30, 45, and 60) on naturally infested cattle	Conidia of *M. anisopliae* × *R. microplus* (L, N and A)	Strain Ma34 and Ma14 (1×10^8^); aqueous suspension (Tween 80 - 0,1 mL)	Efficacies ranged from 0 to 51% (Ma14) and from 0 to 77% (Ma34) throughout the evaluation period.
	Barbieri et al. (2023)	Spraying (1×) on naturally infested cattle	Conidia of *M. anisopliae* × *R. microplus* (L, N and A)	Strain CEPE-26 (5×10^7^): oil-based suspension (Ma01: mineral oil 5%, Ma02: silicon oil 0,01%, mineral oil 2,5%); Triton X-100 (0.02%).	The MaO2 formulation reduced the number of ticks on animals between days +7 and +21 (efficacy: 32–66%), whereas the MaO1 formulation yielded reduction only on day +21 (efficacy: 0–54%).
	Oundo et al. (2024)	Spraying (13× every 14 days for 182 days) on naturally infested cattle	Conidia of *M. anisopliae* x *R. appendiculatus*, *R. decoloratus* and *A. variegatum* (L, N and A)	Strain ICIPE 7(1×10^9^) commercial product (Tickoff – vegetable oil 95%; querosene 3,5% and Triton X-100 1,5%).	The fungal formulation exhibited an efficacy of 72.5%; however, it did not differ statistically from the group treated only with the formulation excipients. The fungal formulation did not reduce the incidence of *T. parva* or *A. marginale.*
	Hidalgo et al. (2025)	Spraying (3×) on naturally infested cattle	Conidia of *B. bassiana* × *R. microplus* (L, N and A)	Strain INIAP L3B3 (1× 10^6^)	Fungal applications reduced the number of ticks on treated animals.
	Zuñiga-Rivera et al. (2025)	Spraying (2× on days 0 and 21) on naturally infested cattle	Conidia of *M. anisopliae* × *R. microplus* (L, N and A)	Strain Ma14 (1×10^4^ e 1×10^2^); diatomaceous earth (0,75% e 0,5%); Tween 80 (0,1%)	Efficacy increased progressively, reaching values above 90% (Ma 1×10^2^ + diatomaceous earth 0.75%) and 80% (Ma 1×10^4^ + diatomaceous earth 0.5%) from day 21 onward, lasting until day 42.
					
**In the environment**	Benjamin, et al. (2002)	Spraying (1×) on naturally infested vegetation	Conidia of *M. anisopliae* × *I. scapularis* (A)	Strain ESC 1 (4×10^9^); commercial product (Bio-Blast Biological Termiticide) diluted in water	Tick mortality in the treated group was 53% (40/76), versus 3% in the control group (3/92).
	Bittencourt et al. (2003)	Spraying (1× per bioassay, it was 3 bioassays) on artificially infested vegetation	Conidia of *M. anisopliae* × *R. microplus* (L)	Strain ESALQ 959 (10^7^ and 10^9^); aqueous suspension (spreading adhesive not specified)	The total reduction rate for the first bioassay was 17.9% and 17.4% for 10^7^ and 10^9^ conidia/ml, respectively; in the second bioassay it was 22.5% and 52.3% for 10^7^ and 10^9^ conidia/mL, respectively; in the third bioassay it was 37.8% and 53.8% for 10^7^ and 10^9^ conidia/mL, respectively.
	López et al. (2009)	Spraying (3× at 7-day intervals) on naturally infested vegetation	Conidia of *M. anisopliae* × *R. microplus* (L)	Strain 137bm (5×10^12^ conidia/ha); formulation with lactose, talc and xanthan gum (concentrations not reported)	The fungal formulation caused an 86% reduction in the larval population one week after treatment.
	Ángel-Sahagún et al. (2010)	Spraying (1×) on naturally (first trial) and artificially (second trial) infested vegetation	Conidia of *M. anisopliae* × *R. microplus* (L)	Strain Ma 14 (2 × 10^9^ CFU/m^2^); 2 aqueous formulations (with Tween 80 0.1% or Citroline 1%) and 2 solid formulations (with wheat bran: 50 kg/ha or Celite: 50 kg/ha)	In the first field trial, conidial formulations in Celite and wheat bran caused 67.8% and 94.2% population reduction, respectively. In the second trial, the Tween formulation caused the highest larval reduction, reaching up to 61%. Wheat bran formulation caused 58.3% larval reduction.
	Stafford e Allan (2010)	Spraying (2×) on naturally infested residential sites (lawn and woodland)	Conidia of *B. bassiana* and *M. anisopliae* × *I. scapularis* (N)	*B. bassiana* strain ATCC 74040 (2.3×10^17^ viable spores of product - 95.5 ml/100 m^2^), commercials products: Naturalis T&O - *B. bassiana* strain GHA (2.2×10^9^ viable spores/100 m^2^) and BotaniGard ES - *M. anisopliae* stain F52 (2.5×10^11^ viable spores/100 m^2^)	All products significantly reduced the number of nymphs in the treated areas compared to the control areas.
	Nchu et al. (2010)	Spraying (3× - day 0, 14 and 28) on artificially infested vegetation associated with semiochemical-baited traps	Conidia of *M. anisopliae* s.l. × *A. variegatum* (A)	Strain ICIPE 07 (1×10^9^); emulsifiable formulation (49.5% sterile distilled water, 49.5% corn oil and 1% Tween 80)	*Metarhizium anisopliae*-treated semiochemical-baited caused a relative tick reduction of 63%.
	Ojeda-Chi et al. (2010)	Spraying (3× - day 0, 14 and 28) on artificially infested vegetation, in the wet and dry season	Conidia of *M. anisopliae* × *R. microplus* (L)	Strain Ma 34 and Ma 14 (1×10^8^); fungal suspension added to wheat bran	In the wet season trial the efficacy was 67.7% and 100% in the dry season trial.
	Garcia et al. (2011)	Spraying (1 × every 21 days, totaling 12 applications) on naturally infested vegetation	Conidia of *M. anisopliae* × *R. microplus* (L)	Strain Esalq E9 (1.37 to 2.20 × 10^7^ viable conidia/ m^2^); aqueous suspension (Tween 80 0.1%)	No deleterious effect of pasture sprayings with fungus over ticks was detected.
	Samish et al. (2014)	Spraying (1 ×) on artificially infested sandy loam soil covered with dry eucalyptus foliage	Conidia of *M. brunneum* × *R. annulatus* (A)	Strain 7 (2×10^7^ conidia/cm^2^); oil suspension (10% canola oil or 10% mineral oil and 0.01% Triton X-100)	There was no hatching of eggs laid by females placed on ground sprayed with conidia in water with canola or mineral oils.
	Nana et al. (2015)	Spraying (1×) on artificially infested vegetation	Conidia of *M. anisopliae* × *R. appendiculatus* (A)	Strain ICIPE 07 (1×10^9^); emulsifiable formulation of *Calpurnia aurea* extract (attracting substance)	Mortality of 83% was observed among field-collected ticks. A significant reduction was recorded in body weight, egg-mass and egg hatchability from fungus-infected females.
	Wassermann et al. (2016)	Spraying (1×) on containers with earth covered by oak/beech foliage	Blastospores of *M. anisopliae* s.l. × *Ixodes ricinus* (N, L)	X-1c (1×10^7^ blastospores/cm^2^); unspecified suspension	Only 18.5% of the larvae were able to develop into nymphs. Only 7.1% of nymphs were recovered as adult ticks after fungal treatment.
	Mesquita et al. (2020)	Spraying (1×) on artificially infested vegetation	Conidia of *M. anisopliae* × *R. microplus* (A, N, L, E)	Strain LCM 04 (1×10^7^ conidia/cm^2^); oil-in-water emulsion (10% mineral oil and 1% Tween 80)	The fungus-treated grass pots had significantly fewer larvae than the control pots.
	Marciano et al. (2020)	Spraying (1×) on artificially infested vegetation	Conidia of *M. anisopliae* s.l. × *R. microplus* (A, N, L, E)	Strain CG 148 and CG 347 (2×10^7^ conidia/cm^2^); mineral and vegetable oil emulsions (10% mineral or vegetable oil and 1% Tween 80)	The emulsion containing CG 148 and vegetable oil resulted in an average tick control efficacy of 90%, while the CG 347 formulation in mineral oil achieved 96%.
	Marciano et al. (2021)	Spraying (1×) on artificially infested vegetation	Microsclerotia and blastospores of *M. robertsii* × *R. microplus* (A, N, L, E)	Strain IP 146 (0.5 and 0.25 mg/cm^2^); granular formulation (microcrystalline cellulose and Psyllium)	Relative efficacy (first evaluation) of 38% (blastospores) and 65% (microsclerotia).
	Schulze, Jordan (2021)	Spraying (3×) on naturally infested vegetation	Conidia of *M. anisopliae* x *I. scapularis* and *Amblyomma americanum* (N)	Strain F52 (10.6 ml AI/plot); commercial product Met52 EC Bioinsecticide (11% *M. anisopliae*)	Met52 EC Bioinsecticide failed to maintain a 90% suppression threshold for *I. scapularis*, and required two additional applications over the trial.
	Almeida et al. (2022)	Spraying (1× every 30 days for 5 months) on artificially infested vegetation	Conidia of *M. anisopliae* × *R. microplus* (A, N, L, E)	Strain IBCB 425 (2 × 10^13^ conidia/hectare); solution of water and mineral oil in the proportions of 19:1	The average treatment efficacy ranged from 68.91 to 90.35 and the mean percentage inhibition of oviposition ranged from 35.64 to 64.37 over five months.
	Santos et al. (2022)	Spraying (1× every 30 days for 5 months) on naturally infested vegetation	Conidia of *M. anisopliae* × *R. microplus* (A, N, L, E)	Strain IBCB 425 (1 × 10^13^ conidia per hectare); solution of water (95%) and mineral oil (5%)	The annual mean efficacy of the treatments was 36% and 48% for two different areas in the study.
	Pereira et al. (2022)	Spraying (1×) on artificially infested vegetation	Conidia of *M. anisopliae* × *R. microplus* (A, N, L, E)	Strain ARSEF 3643 (2.0 × 10^8^ conidia/mL); aqueous formulation (0.1% Twenn 80) and oil formulation (10% mineral oil and 0.1% Twenn 80)	Oil formulation of *M. anisopliae* s.l. and aqueous formulation yielded a tick control percent between 58.4% and 61.3% and 39.6% and 43.8%, respectively.
	Pereira et al. (2024)	Spraying (1× every 30 days for 5 months) by drone on naturally infested vegetation	Conidia of *P. lilacinum* x *R. microplus* (A, N, L, E)	Strain of IBCB 130 (1.2 × 10^12^ conidia per hectare); 5 g of *P. lilacinum* in 200 g of wheat flour	The treatment had a mean efficacy of 48.59%. December and January exhibited the highest efficacy rates: 63.50% and 83.87% respectively.
	Filgueiras et al. (2025)	Spraying (1×) on artificially infested vegetation	Microsclerotia of *M. robertsii* × *R. microplus* (A, L, E)	Strain IP146 (3.75×10^2^ microsclerotia/g, 10 g/plot); granular formulaion, combined or not with entomopathogenic nematode *H. bacteriophora*	Field applications during the rainy season achieved tick population reductions of 54.09% (*M. robertsii*), 38.11% *(H. bacteriophora*), and 46.72% (combination). During the dry season, only the fungal formulation significantly reduced tick populations (26.27% efficacy).
	Andrade et al. (2025)	Spraying (3× at 21-day intervals) by drone on naturally infested vegetation	Conidia of *M. anisopliae* × *R. microplus* (A, N, L, E)	strain IBCB 425 (4 × 10^13^ conidia/ha); fungal dry powder	The fungal biological control (FMT) achieved tick reduction comparable to chemical methods (CCT). FMT milk samples contained no detectable residues, while CCT milk contained chlorpyrifos, cypermethrin, and piperonyl butoxide within 7 days after application.

Entomopathogenic fungi: *Metarhizium*(*M.*); *Beauveria* (*B.*)*; Purpureocillium* (*P.*). Ticks: *Rhipicephalus* (*R*.); *Dermacentor* (*D*.); *Amblyomma* (*A*.); *Ixodes* (*I*.). E: Eggs; L: Larvae; N: Nymphs; A: Adults. ( ) – Fungal concentration used.

The use of adjuvants, including mineral, vegetable, and silicon oil, has enhanced the efficacy of field studies aimed at tick control (Camargo et al., 2016; Zeina et al., 2022). This improvement is associated with enhanced adhesion of conidia to the tick cuticle, which facilitates fungal germination and penetration into the target arthropod (Angelo et al., 2010; Camargo et al., 2012). In addition, adjuvants contribute to protecting the fungus from stressful abiotic factors such as low humidity, high temperatures, and ultraviolet radiation (Barreto et al., 2016; Fernandes et al., 2015; Muniz et al., 2020). This protection against abiotic stress factors becomes even more relevant when fungi are applied directly to cattle, an environment that differs substantially from the natural habitats where EPF typically occur. Such conditions likely expose the fungi to greater environmental stress, thereby emphasizing the importance of protective formulations and adjuvants to maintain their viability and infectivity. Moreover, it is important to observe that the adjuvants used in these studies are not merely inert components; they also exhibited direct toxic effects on ticks in the used concentrations (Camargo et al., 2014; Barbieri et al., 2023), which undoubtedly contributes to the enhanced efficacy observed.

Most field and semi-field studies using EPF for tick control have been conducted with fungi of the genus *Metarhizium*, which has been identified as the most promising group for this purpose (Beys-da-Silva et al., 2020; Monteiro et al., 2024). However, studies using fungi of the genus *Beauveria* have also been reported, showing favorable results (Fernandes et al., 2008; Monteiro et al., 2024). Fungi of the genus *Cordyceps* have attracted increasing interest in Brazil in recent years due to their potential for arthropod biocontrol in crop protection (Monteiro et al., 2024; Mascarin et al., 2024). Although studies have already demonstrated their pathogenicity against ticks under laboratory conditions (Angelo et al., 2012), no field evaluations have been conducted to date. These and other fungi that exhibit potential but have not yet been tested under field conditions should be considered in future research efforts. Regarding tick species, most studies have focused on species of the genus *Rhipicephalus* (Alonso-Díaz et al., 2007; Kaaya et al., 2011; Rot et al., 2013; Murigu et al., 2016a), particularly *R. microplus* (Alonso-Díaz et al., 2007; Bahiense et al., 2007; Camargo et al., 2016; Zeina et al., 2022; Zuñiga-Rivera et al., 2025). The stronger research focus on this species is due to its major relevance to global cattle production, as *R. microplus* has a broad geographic distribution and causes substantial economic losses in livestock systems (Martins et al., 2006; Grisi et al., 2014; Rodriguez-Vivas et al., 2018).

All field studies to date have been conducted using aerial conidia (Table 1); however, promising results have been reported with the use of *Metarhizium* spp. and *Beauveria* spp. blastospores against engorged females of *R. microplus* under laboratory conditions (Bernardo et al., 2018), highlighting the need for field evaluations employing this type of propagule. Most studies have used a concentration of 10^8^ conidia/mL, with a few exceptions that employed concentrations of 10^7^ conidia/mL (Barbieri et al., 2023), 10^6^ conidia/mL (Hidalgo et al., 2025), 10^2^ and 10^4^ conidia/mL (Zuñiga-Rivera et al., 2025), showing a broad range of concentrations evaluated across different studies with interesting results.

Studies involving the application of EPF on animals have demonstrated their potential to contribute to tick control; however, the number of such studies remains limited compared to extensive research conducted under laboratory conditions. To advance this field, efforts must be directed toward conducting field trials. Despite recent progress, some aspects still require further investigation, including: (i) assessing the efficacy of different fungal propagules for tick control; (ii) evaluating the performance of fungal treatments in cattle of different breeds; (iii) conducting long-term studies that implement integrated management strategies combining fungal applications with other control technologies; (iv) assessing whether fungal treatments can replace chemical acaricides in one or more tick generations, reducing chemical use; (v) comparing fungal efficacy using different spraying devices; (vi) performing trials with lower fungal and adjuvant concentrations to improve the economic feasibility of the technology; and (vii) assessing whether the application time influences treatment efficacy time of the day or season).

### Off-host application

Field applications of EPF on the soil or over the pasture to control non-parasitic phases [eggs, larvae, nymphs (depending on the tick species), and adults] of ixodid ticks, under field or semi-field conditions, have been reported in scientific studies since 2002 (Table 1). The first study reported the use of a commercial product based on *M. anisopliae* against adults of *I. scapularis* (Benjamin et al., 2002). This study was conducted with high conidial concentration (4 × 10^9^ conidia/m applying 1-1.5 liters per 100m^2^). The treatment yielded 53% mortality of ticks.

In 2003, a study was conducted using grass plots artificially infested with *R. microplus* larvae (Bittencourt et al., 2003). The authors tested the efficacy of *M. anisopliae* conidial suspension at 10^7^ or 10^9^ conidia/mL applying 60 mL per m^2^. The highest tick control efficacy (53.8%) was observed in the third bioassay. This study did not use any adjuvant for the conidial suspension, although entomopathogenic fungal propagules are rarely applied in the field as unformulated mycoinsecticides in agricultural crops, as adjuvants can protect them against detrimental abiotic factors, extend their field persistence, and increase their infectivity (Paixão et al., 2017). Accordingly, the same applies to tick control in the field.

More recent studies used a variety of different adjuvants, mostly mineral or vegetable oil-in-water emulsions, to enhance fungal performance (Ángel-Sahagún et al., 2010; Nchu et al., 2010; Ojeda-Chi et al., 2010; Samish et al., 2014; Mesquita et al., 2020; Marciano et al., 2020; Schulze & Jordan, 2021; Almeida et al., 2022; Pereira et al., 2022; Santos et al., 2022) (Table 1). Two studies also reported the use of solid CO_2_ and semiochemical-baited or plant-extract-baited traps coupled with the fungal treatment (Nchu et al., 2010; Nana et al., 2015). As important as the use of a proper adjuvant or the use of baited traps, the capacity in the production of propagules is also essential because the required concentration of fungi to control the ticks in the field should be high. Hard ticks are naturally much less susceptible than insects to entomopathogenic fungi (Ment et al., 2012). Most studies involving the field use of EPF against ixodid ticks used aerial conidia as the fungal propagule, although recent field studies reported the successful use of other EPF propagules produced in submerged liquid culture (i.e., blastospores and microsclerotia) against ticks (Wassermann et al., 2016; Marciano et al., 2021, Filgueiras et al., 2025).

Most field studies using fungi on the vegetation to control ixodid ticks were conducted with *Metarhizium* isolates (Table 1), except Stafford & Allan (2010) that used *Beauveria* isolates and Pereira et al. (2024) that used *P. lilacinum* isolate. The latter and Andrade et al. (2025) used a drone to apply the fungus to pasture. Among the studies that treated the environment (not the host) here analyzed, the majority involved *Rhipicephalus* ticks (more frequently *R. microplus* ticks), followed by *Ixodes* ticks (more frequently *I. scapularis*) and *Amblyomma* ticks (one study with *A. variegatum* and one with *A. americanum*) (Table 1).

EPF applied in the environment can negatively impact tick larval outbreaks even when fungal formulations are not applied directly to the tick larvae (Mesquita et al., 2020; Marciano et al., 2020; Pereira et al., 2022; Filgueiras et al., 2025). This confirms the importance of the environmental persistence of an EPF. The levels of environmental persistence of an EPF can be supported by the fungus-plant endophytic interaction and by the fungal rhizosphere competence. On the other hand, a series of abiotic obstacles, such as low humidity, high incidence of sunlight, rain, and high temperatures can also negatively affect EPF environmental persistence. Accordingly, several variables can be involved in the success of field use of fungi against ticks including, but not only, fungal species and isolate (and their virulence to ticks and tolerances to abiotic factors), type of propagule, presence or absence of adjuvants, type of vegetation, and soil nature.

The use of EPF in field applications has been shown to be safe and effective. While many studies focused on conidial application directly to animals and pasture, drone-based strategies represent a promising next step for integrating EPF into livestock tick management. However, there is still a lack of information concerning i) comparison between the different types of fungal propagules (regarding their virulent effects against tick populations in the field or tolerance to abiotic factors); ii) possible interactions between EPF and the vegetation/soil, and what is the impact for fungal environmental persistence; iii) number and frequency of fungal applications to hold tick outbreaks; iv) the impact of field fungal applications in the incidence of tick-borne diseases; v) comparison of the efficacy of soil applications under different biomes and climatic conditions.

### Economic analysis and cost-benefit assessment

In the Brazilian market, there are currently no commercial products based on EPF for tick control, which makes it difficult to estimate the treatment cost per animal. However, in the development of technologies for tick control, two aspects should be considered: (1) the treatment cost per animal will likely be higher compared to the current market cost of chemical acaricides, especially those based on spraying strategies; and (2) the adoption of biological control technologies will reduce the odds of chemical residues in food and the environment.

Most chemical acaricides based on spray delivery currently available on the market cost between 0.2 and 0.8 USD per animal. This estimate refers only to spray acaricides, which are recommended for use in lactating cows, a market segment where fungal-based products would likely experience greater demand and acceptance among producers. For pour on and injectable acaricides, higher prices are generally observed, ranging from 1.1 to 6.5 USD per animal for most formulations available on the market, and reaching up to 14.8 USD (recently introduced products - fluralaner as the active ingredient). In this sense, future EPF-based products may represent an interesting alternative for farms with high levels of multiresistance to acaricides, which in some cases largely rely on acaricides formulations with high costs, such as isoxazolines.

Reducing fungal concentrations in field trials is a desirable approach that should be considered in future studies to improve the economic feasibility of these products for tick control, since most field studies use concentrations of 10^8^ conidia/mL. In this context, some studies have explored the use of lower concentrations, such as 10^7^ conidia/mL (Barbieri et al., 2023), 10^6^ conidia/mL (Hidalgo et al., 2025), and 10^2^ or 1 × 10^4^ conidia/mL (Zuñiga-Rivera et al., 2025). Barbieri et al. (2023) estimated a treatment cost of USD 2 per animal, indicating that lower fungal concentrations may contribute to improved economic feasibility. This estimate was based on a prototype and considered only the raw material cost for a single unit. Although large-scale production may optimize conidia production costs, other aspects must be considered, since additional expenses will be involved, such as specialized labor, plant maintenance, inert and adjuvant formulation components, packaging costs, storage and transport logistics, profit margins for both the company and the retailer, production losses, and taxes. Anyway, considering the added value of the technology (discussed later), it gives an optimistic scenario for a potential cost of future EPF-based products.

In field studies, adjuvants were used in concentrations between 2.5 and 20% (10% was the most used concentration) (Table 1). Considering two main factors: (i) the price of adjuvants (mineral and vegetable oils) available in the Brazilian agricultural market, and (ii) the use of 5 L of spray mixture for the treatment of adult cattle; the inclusion of these adjuvants may lead to additional costs in the treatment per animal which may range from 0.46 USD (2.5% concentration) to 8.7 USD (20%), with an intermediate cost of approximately 4.4 USD at a 10% concentration. Future studies combining fungi and adjuvants for tick control should prioritize the determination and use of the low oil concentrations which are still effective but aiming at cost reduction and economic feasibility. Accordingly, concentrations already used for crop protection may help guiding this approach.

Another aspect that should be considered in the adoption of biological acaricides for tick control (application on the host) is the treatment interval. For example, within strategic tick control programs for *R. microplus*, spray-based chemical acaricide treatments are generally applied at intervals of 21 to 28 days, allowing infestation levels to be maintained without compromising animal health and productivity (Labruna, 2008; Nicaretta et al., 2023; Leal et al., 2024). For pour-on or injectable products, longer treatment intervals are generally observed, ranging from 35 to 80 days (Nicaretta et al., 2021; Aquino et al., 2024). In field studies involving EPF with extended experimental durations, treatment intervals have ranged from 7 to 28 (7, 15, 21 and 28) days (Lopez et al., 2009; Murigu et al., 2016b; Webster et al., 2015; Alonso-Díaz et al., 2022; Oundo et al., 2024). In short-term studies, more than one application was reported during the initial days (Zeina et al., 2022; Camargo et al., 2016). Considering (i) economic feasibility and (ii) treatment interval of spray chemical acaricides, it is recommended that future studies adopt treatment intervals of 21 or 28 days. Considering environmental applications, a tentative approach would be to perform treatments every 30 days (Pereira et al., 2024), although shorter intervals of 21 days may also be considered (Andrade et al., 2025). It is important to emphasize that in such studies, tick reduction should be evaluated not only in the pasture but also on the animals grazing in these areas.

In this cost–benefit balance, other aspects should be considered regarding the value of these biological products for tick control, considering factors aligned with the concepts of sustainability and One Health. Among these aspects, the following can be highlighted: environmental safety, through reduced impact on non-target organisms and residue-free environments; food safety, by ensuring the production of residue-free animal products; occupational safety, through the use of organisms that pose no intoxication risk to humans; and animal safety, through the application of products that do not pose toxicity risks to cattle. In addition, treatment with biological products may also reduce the number of chemical treatments required per year, thereby slowing the development of acaricide-resistant tick populations.

These aspects can contribute to more resilient and sustainable production systems. In the future, the adoption of biological acaricides with EPF may add value to products derived from cattle farming through specific certifications and access to more demanding export markets. Therefore, the economic assessment of these technologies should consider not only the cost and immediate financial return but also the added value to production and the ecological and social benefits associated with their adoption.

## Regulatory Framework and Safety Assessment

In most countries, EPF are regulated under pesticide legislation and classified as microbial biopesticides. Despite their generally safer toxicological profile, they must meet similar regulatory standards as chemical pesticides. Agencies such as the Environmental Protection Agency - EPA (USA), European Food Safety Authority - EFSA (EU), and the Ministry of Agriculture and Livestock (Ministério da Agricultura e Pecuária - MAPA) (Brazil) oversee registration, often in collaboration with others like the Brazilian National Health Surveillance Agency (Agência Nacional de Vigilância Sanitária - ANVISA), Brazilian Institute of Environment and Renewable Natural Resources (Instituto Brasileiro do Meio Ambiente e dos Recursos Naturais Renováveis – IBAMA), the USA Food and Drug Administration (FDA), the United States Department of Agriculture (USDA), and national bodies within the EU. These multi-agency frameworks ensure thorough evaluations of efficacy, human health, and environmental safety. However, the process remains complex and time-consuming, limiting broader adoption.

The regulation of entomopathogenic fungi-based products goes beyond registration; legislation also affects the collection, transportation, intellectual property rights, and risk classification of these microorganisms, directly influencing access, innovation, and the safe use of biological agents (Smith et al., 2023). A reassessment of regulatory frameworks is often necessary, aiming for greater flexibility and alignment with the specific characteristics of microbial products. Humber (2016) argues that while the commercially used EPF may fit existing regulatory and business models, legal and commercial constraints have unnecessarily, and perhaps unwisely, limited the diversity of biocontrol fungi in use. This has led to a narrow and overly stable list of broad-spectrum species, which raises concerns about non-target effects and overlooks the potential of highly specific and safer alternatives.

In this scenario, EPF are widely recognized as safe biological control agents for both human and environmental health when properly characterized and applied (Zimmermann, 2007; OECD, 2008; Buonsenso, 2025). Studies conducted by the USDA and independent laboratories have consistently shown that these fungi exhibit no pathogenic or toxic effects on mammals, birds, fish, and beneficial arthropods under normal exposure conditions (EPA 2012). Acute oral and dermal safety studies in rodents and rabbits are typically performed at doses several orders of magnitude higher than those that could realistically occur under environmental or field exposure conditions (EPA 2011; 2018; 2020). These tests consistently report no clinical signs of toxicity, mortality, or tissue alterations, even at extremely elevated concentrations. This outcome provides a strong safety margin: if no adverse effects are observed under such exaggerated exposure scenarios, it can be reasonably inferred that lower concentrations, those used in practical agricultural or veterinary applications, pose no toxicological risk. Therefore, while these high-dose studies represent a conservative approach intended to ensure biosafety, their results also reinforce the intrinsic non-toxic nature of EPF, confirming that their real-world use is unlikely to produce harmful effects on mammals or other non-target organisms. Similarly, field trials involving livestock treated with *Metarhizium*-based formulations have not demonstrated adverse behavioral or physiological effects (Camargo et al., 2016; Barbieri et al., 2023).

In agricultural risk assessment, EPF products undergo toxicological and ecotoxicological testing comparable to those required for microbial pesticides. These include acute and sub-chronic oral, dermal, and inhalation studies in mammals, as well as non-target organism assays involving pollinators (*Apis mellifera*), aquatic species, soil invertebrates, and beneficial predators (OECD, 2008; Brasil, 2023).

An additional natural safety factor arises from the self-grooming (auto-cleaning) behavior of insects, especially social insects that are beneficial (pollinators and parasitoids), which substantially reduces the likelihood of non-target infection. Grooming effectively removes conidia that are not strongly adhered to the cuticle, thereby limiting transmission and reducing the persistence of fungal propagules on non-target hosts (Reber et al., 2011; Shang et al., 2015; Casillas-Pérez et al., 2023). Together, the combination of biological specificity, low mammalian toxicity, environmental degradability, and behavioral barriers supports the strong biosafety profile of EPF used in both agricultural and veterinary contexts.

The rising demand for sustainable agricultural solutions has expanded interest in biological products for both crop and animal health. To reach the market, these products must comply with regulations that guarantee safety for animals, the environment, and users. Globally, countries are moving toward specific legislation for microbial products. France, Brazil, Argentina, and Colombia are examples of countries that are leading this shift through public policies and national programs that support bioinputs (Goulet, 2021; Medina et al., 2024; AgroPages, 2025a). In addition, countries such as Pakistan, New Zealand, Mexico, South Africa, and Japan have also advanced regulatory frameworks or initiatives aimed at facilitating the development and commercialization of biologicals (AgroPages, 2025b; A Lighter Touch, 2025; Mexico Business News, 2025; SentonPharm, 2025). These efforts demonstrate a global trend toward harmonizing regulatory pathways and promoting the adoption of biopesticides and bioinputs as viable alternatives to conventional chemical products in both agriculture and livestock production.

The global landscape for registered products using EPF for tick control remains limited and fragmented, despite the strong potential demonstrated in research (Camargo et al., 2016; Sullivan et al., 2022; Oundo et al., 2024). Products specifically developed for ticks may have unstable market presence, as seen with Met52® in the United States. Commercialization of EPF for tick control is hindered by regulatory and market challenges, with regional disparities in product availability and a focus on other agricultural pests. Complex and inconsistent regulations across countries, combined with the need for robust field validation, slow the adoption of these fungi as viable, widely accessible alternatives to chemical acaricides.

In Brazil, veterinary products are regulated by MAPA. For EPF intended for use in animals, the regulatory pathway is not yet fully harmonized, creating a discrepancy. Depending on the intended use and claims, the product may be classified as a pharmaceutical antiparasitic (governed by MAPA’s Technical Regulation for antiparasitics, Ordinance 48/1997) (Brasil 1997) or as a veterinary biological (under the framework of biologicals, traditionally applied to vaccines and immunobiologicals, e.g., Ordinance 74/1996) (Brasil, 1996). This distinction has significant implications. As an antiparasitic, the product must meet requirements aligned with pharmacological safety and toxicology testing, also presenting more than 95% of efficacy, which is difficult to be achieved by a biological product. In the regulatory framework for veterinary biological products, efficacy is no longer the main challenge; however, these products follow a regulatory structure similar to that applied to vaccines, with a strong emphasis on microbial identity, biosafety, and immunobiological production standards. This framework imposes stringent requirements substantially increasing production costs. This is particularly critical when the same manufacturing facility is developed to produce both vaccines and fungal propagules. MAPA requires strict segregation of processes, risk assessment of cross-contamination, and validated cleaning and biosafety protocols. Choosing one pathway or the other affect dossier structure, facility approval, labeling, pharmacovigilance obligations, and even how inspections are carried out. In practice, companies often seek prior consultation with MAPA’s veterinary products coordination to align expectations before formal submission.

At the broader policy level, the approval of the bioinputs Law No. 15,070 of 2024 (Brasil, 2024) created a national legal framework for the production, registration, commercialization, use, and disposal of bioinputs in agriculture, livestock, aquaculture, and forestry. Although this law does not replace MAPA’s specific rules for veterinary products, it provides strategic support for innovation, R&D, and regulatory recognition of biological control agents, including those used against ectoparasites in livestock. Its provisions influence how regulators, companies, and producers perceive classification, experimentation, labeling, waste management, and sustainable integration of biologicals into production chains. Following the publication of the law, and under the coordination of the MAPA, the regulatory process of the referred legislation is currently underway. This process involves the participation of various professional societies and unions representing different stakeholders within the production chain. After the regulation is finalized, the normative instruments for product registration will be structured or revalidated (for the existing regulations). All these efforts aim to establish clear criteria and regulatory pathways that enable these innovations to reach the market, thereby promoting advances in agricultural and livestock production in Brazil.

In parallel, the Brazilian College of Veterinary Parasitology (Colégio Brasileiro de Parasiotlogia Veterinaria - CBPV), a scientific society that brings together researchers, professionals, and students working in the field of veterinary parasitology in Brazil, has been discussing the need for specific guidelines for the registration of biological acaricides. While CBPV has not yet published an official guideline, its role as a scientific corporation suggests it may contribute to standard-setting, in cooperation with MAPA and other associations. Such guidelines would help clarify requirements for efficacy trials, safety testing, and dossier preparation, complementing the current legal framework and reducing uncertainty for developers. It may serve as a stimulus for other scientific societies worldwide to take part in the development and discussion of guidelines, thus providing high-qualified suggestions for the society.

In conclusion, the regulatory approval of acaricides based on EPF globally depends not only on demonstrating efficacy and safety but also on navigating the current regulatory ambiguity between the pharmaceutical and biological pathways. The Brazilian bioinputs law provides initial legitimacy for biological control strategies and could serve as a reference for other countries to develop their own livestock bioinputs regulations or even adopt Brazil’s framework (once finalized) where regulatory convergence allows. While initiatives like the CBPV’s efforts to draft specific guidelines may help in regulatory gaps. Together, these movements point toward a more harmonized, consistent and predictable regulatory environment, which could accelerate the adoption of sustainable biological acaricides in veterinary practice.

## Knowledge Gaps and Future Research Directions

Research conducted on the use of EPF for tick control, over the past few years, has increasingly demonstrated that these microorganisms represent highly promising alternatives for the integrated and sustainable management of tick infestations. These results are due to the physiological characteristics of these microorganisms, which enable them to penetrate directly through the arthropod cuticle, as well as to their biotic traits and environmental requirements for survival and maintenance, conditions that closely overlap with those needed by the target arthropods in their natural habitats.

Recent advances have significantly expanded our understanding of tick immune responses against EPF, revealing a complex and multifactorial defense system. Cellular responses mediated by hemocytes, including phagocytosis, melanization, and cytotoxic interactions with fungal structures, together with humoral mechanisms involving complement-like proteins, antimicrobial peptides, and redox metabolism, play central roles in limiting fungal development. Despite these advances, major knowledge gaps remain. The molecular signaling pathways underlying hemocyte activation, nodulation, and phagocytosis of EPF are still poorly characterized in ticks, as is the specific contribution of antimicrobial peptides during fungal infections. Moreover, the role of immune priming appears limited or absent, and the dynamics of redox metabolism and reactive oxygen species generation in tick hemocytes during EPF infection remain largely unexplored. Addressing these gaps through integrative molecular, cellular, and functional approaches will be critical for elucidating tick–fungus interactions and for optimizing EPF-based biocontrol strategies capable of circumventing or exploiting tick immune defenses.

**Figure 3:**
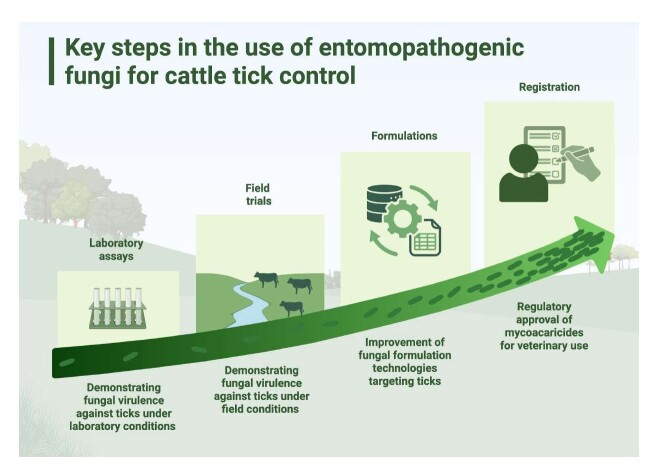
Key steps in the use of entomopathogenic fungi for cattle tick control.

Field studies with ticks indicated that the development of adequate formulations for direct application to animals still requires further research for proper implementation, considering the diversity of proposed use methods and application strategies. Significant progress has been achieved, particularly with EPF combined with adjuvants. Several studies reported that substances that protect and enhance the germination and development of EPF in the environment can and should be incorporated into formulations. In parallel, for field treatment of infested areas, formulations based on microesclerotia designed for soil release, targeting the infection of non-parasitic tick stages present in pastures, are being developed and have yielded highly promising and economically viable results. However, the success of these approaches ultimately depends on regulation of these products for animal health purposes, overcoming formulation challenges, including propagule stability, protection against abiotic stressors, and compatibility with livestock production systems. Addressing these bottlenecks will be essential to move EPF-based tick control from experimental trials toward practical, large-scale application (Figure 3).

In Brazil, however, despite decades of research, the same regulatory framework established by MAPA remains in effect for the approval of tick control products for veterinary use. These standards, defined by MAPA Ordinance No. 48/1997, require a 95% efficacy threshold for the licensing and registration of acaricidal products intended for use in infested animals. Such requirements have limited the industry’s interest in biological products, as it is impractical to invest in the development of a biological product that would likely not reach the high efficacy rates demanded for chemical acaricides (95%) and would therefore face challenges in obtaining marketing authorization. To align regulatory norms with the new reality of biological products, committees have been established within CBPV and MAPA to propose updates. Additionally, the requirement that biological products based on EPF be manufactured in facilities meeting the same regulatory standards required for vaccine production represents another major challenge.

In a scenario of widespread acaricide multi-resistance, it is timely to question whether maintaining a rigid 95% efficacy threshold is appropriate for the registration and commercialization of non-chemical control products intended as alternatives to conventional acaricides. Field evidence indicates that several chemical compounds currently available on the market, particularly pyrethroids, frequently fail to achieve this level of efficacy under practical farming conditions. Moreover, in herds exhibiting high levels of multi-resistance (often involving resistance to six out of seven acaricidal classes) (Klafke et al., 2024) it is not uncommon for the most effective conventional products, except for isoxazolines, to reach only 60–70% efficacy. In such contexts, the availability of a non-chemical product with no withdrawal period that consistently achieves 50–70% efficacy represents a valuable and pragmatic tool within integrated tick management programs, contributing to resistance mitigation, reduced chemical pressure, and improved sustainability of control strategies.

Another significant limiting factor for the advancement of biological products is the perception of livestock producers regarding tick control. Many farmers still expect immediate results and 100% efficacy, typical of conventional synthetic acaricides, which hinders the acceptance of biological solutions whose effects are gradual and dependent on environmental conditions. However, it is important to note that a considerable proportion of livestock producers in Brazil are also grain producers, who already use and trust biological control agents in agricultural crops. This dual activity represents a strategic opportunity to accelerate the adoption of biological products in animal health, by transferring the knowledge and confidence gained from agricultural applications to livestock systems.

In this way, to overcome current barriers, a coordinated effort among government, industry, and research institutions is essential to invest in technical training and communication programs, demonstrating the economic and environmental advantages of biological control. Only through awareness and perception change, supported by practical examples and cross-sector integration, will it be possible to consolidate the use of biological agents as effective tools in integrated tick management.

The advancement of EPF as biological control agents depends not only on scientific validation but also on the establishment of security frameworks. Evidence accumulated over decades confirms that EPF are safe, environmentally compatible, and non-toxic to humans, animals, and beneficial organisms when correctly characterized and applied. The combination of biological specificity, ecological degradability, and natural behavioral barriers such as insect self-grooming reinforces the strong biosafety profile of these fungi.

Furthermore, strategic integrated control programs that combine management practices with both chemical and biological approaches must be developed. These programs should be adapted to the ecological context in which they will be implemented, considering the tick species involved, their distinct biological and behavioral characteristics, the production system, and the geographical and climatic conditions of each region.

## Concluding Remarks

Taken together, the evidence discussed in this review highlights that the development and regulatory approval of EPF for tick control represent not only scientific and technological challenges, but also a regulatory challenge involving governmental agencies and private companies, as well as a strategic necessity for livestock production systems, particularly in tropical regions. Among the most compelling reasons to pursue biological products is the absence of a withdrawal period, a critical advantage that none of the currently available efficacious synthetic acaricides can offer. This limitation directly constrains intensive livestock systems, especially high-yield dairy production. In this context, biological products that provide moderate but consistent efficacy without compromising food safety represent a paradigm shift, enabling continuous production while reducing chemical pressure and resistance selection. The consolidation of EPF-based tools within integrated tick management programs, supported by appropriate regulatory frameworks, formulation advances, and producer awareness, is essential to unlock sustainable productivity gains and to reconcile animal health, food safety, and environmental stewardship in tropical livestock systems. Finally, although less extensively investigated, the available examples in the literature also indicate that the use of EPF may represent a useful tool for the control of ticks that are vectors of human pathogens.

## Data Availability

This article presents a synthesis of existing literature. No original data were produced, and therefore no datasets are available.
